# Supplementary material for: The cellular response to extracellular vesicles is dependent on their cell source and dose
Source: Sci Adv. 2023 Sep 1;9(35):eadh1168. doi: 10.1126/sciadv.adh1168 (PMC11629882; doi:10.1126/sciadv.adh1168)
Supplement: 20230901-1 [file sciadv.adh1168.v1.pdf]

CELL BIOLOGY

Copyright © 2023 The Authors, some rights reserved; exclusive licensee American Association for the Advancement of Science. No claim to original U.S. Government Works. Distributed under a Creative Commons Attribution License 4.0 (CC BY).

The cellular response to extracellular vesicles is dependent on their cell source and dose

Daniel W. Hagey[1,2]*, Miina Ojansivu[3]†, Beklem R. Bostancioglu[1,2]†, Osama Saher[1,2,4], Jeremy P. Bost[1,2], Manuela O. Gustafsson[1,2], Roberto Gramignoli[1], Mathias Svahn[5], Dhanu Gupta[1,2,6], Molly M. Stevens[3,7], André Görgens[1,2,8]‡, Samir EL Andaloussi[1,2]‡

Extracellular vesicles (EVs) have been established to play important roles in cell-cell communication and shown promise as therapeutic agents. However, we still lack a basic understanding of how cells respond upon exposure to EVs from different cell sources at various doses. Thus, we treated fibroblasts with EVs from 12 different cell sources at doses between 20 and 200,000 per cell, analyzed their transcriptional effects, and functionally confirmed the findings in various cell types in vitro, and in vivo using single-cell RNA sequencing. Unbiased global analysis revealed EV dose to have a more significant effect than cell source, such that high doses down-regulated exocytosis and up-regulated lysosomal activity. However, EV cell source–specific responses were observed at low doses, and these reflected the activities of the EV's source cells. Last, we assessed EV-derived transcript abundance and found that immune cell-derived EVs were most associated with recipient cells. Together, this study provides important insights into the cellular response to EVs.

INTRODUCTION

Extracellular vesicles (EVs) are lipid membrane-enclosed structures associated with diverse biologically active molecules. The transfer of these renders EVs important mediators of intercellular communication and has brought attention to their therapeutic and diagnostic potential (*1*–*4*). EVs are secreted by all cells, but their protein (*5*) and nucleic acid (*6*, *7*) cargo varies greatly dependent on their producer cell and mechanism of biogenesis (*8*). This diversity of cargo can impart varied functions on EVs from different cell sources, such as specific cell targeting (*9*, *10*) or unique signaling responses, although the mechanisms controlling this remain unclear (*11*). A second poorly described aspect is the relationship between dose and physiological responses, which is critical to the clinical outcome of EV-based therapeutics (*12*, *13*). This is evident from various studies where bimodal or inverse dose-dependent effect has been observed upon EVs treatment (*14*–*20*). The doses of EVs currently used for in vitro assays are relatively high compared to their physiological levels (*14*, *21*).

RESULTS

To judge the common and specific cellular responses to EVs from different cell sources, we devised an experimental set up whereby human fibroblasts were left untreated, incubated with storage buffer (*22*), or treated with EVs for 24 hours before analysis by RNA sequencing (Fig. 1A) (*23*). Although the role of EVs in immune cell processes are well established (*24*, *25*), fibroblasts were used since they represent a common primary human cell type that is receptive to treatment with EVs (*26*). We analyzed EVs from 12 cell sources, which were separated into five groups of similar cell types: BJ-5ta fibroblasts, bone marrow, umbilical cord blood, and Wharton's jelly–derived mesenchymal stem cells (MSCs) were classified as MSC/fibroblasts. Human umbilical cord endothelial cell (HUVEC) and Panc-1 cell lines were grouped as epithelial cells. Human embryonic kidney (HEK) 293T adherent and HEK293 freestyle suspension cells were the same cell type under different growth conditions. Human amniotic epithelium (HAEC) and CEVEC's Amniocyte Production (CAP) amnionic fluid cells were associated as being of in utero origin. Last, THP1 monocyte and Jurkat "T cell" lines were labeled as originating from immune cells. To confirm the presence of EVs, multiplex bead–based flow cytometry analysis of the EV-associated tetraspanins CD9, CD63, and CD81 was performed, while nanoparticle tracking was used to determine their concentrations (Fig. 1B and fig. S1A) (*27*). Five thousand fibroblasts were then plated and treated with 20, 2000, or 200,000 particles per cell (10^5, 10^7, or 10^9 total particles per well). To assess the effects of these treatments, the cells were imaged under a light microscope and analyzed by flow cytometry to evaluate cell viability. This revealed no gross morphological changes or toxic effects of any of the EVs used (Fig. 1C and fig. S1, B and C).

Because of the myriad different pathways potentially regulated by EVs, we took an unbiased and global approach to assessing their effects on responder cell transcriptomes. Thus, all samples were mapped together using a t-stochastic neighbor embedding–nearest neighbor (tSNE-NN) strategy based on the most variably expressed genes across the dataset (*28*). Although the resulting map agglomerated samples together, regions could be identified, such that all untreated and storage buffer–treated samples clustered closely together (Fig. 1D). To quantify whether this grouping was based on the dosage or cell source of the EVs cells were treated

[1]Department of Laboratory Medicine, Karolinska Institute, Stockholm, Sweden. [2]Department of Cellular Therapy and Allogeneic Stem Cell Transplantation (CAST), Karolinska University Hospital Huddinge and Karolinska Comprehensive Cancer Center, Stockholm, Sweden. [3]Department of Medical Biochemistry and Biophysics, Karolinska Institute, Stockholm, Sweden. [4]Department of Pharmaceutics and Industrial Pharmacy, Faculty of Pharmacy, Cairo University, Cairo, Egypt. [5]NextCell Pharma AB, Stockholm, Sweden. [6]Department of Paediatrics, University of Oxford, Oxford OX3 7TY, UK. [7]Department of Materials, Department of Bioengineering, Institute of Biomedical Engineering, Imperial College London, London, UK. [8]Institute for Transfusion Medicine, University Hospital Essen, University of Duisburg-Essen, Essen, Germany.
*Corresponding author. Email: daniel.hagey@ki.se
†These authors contributed equally to this work.
‡These authors contributed equally to this work.

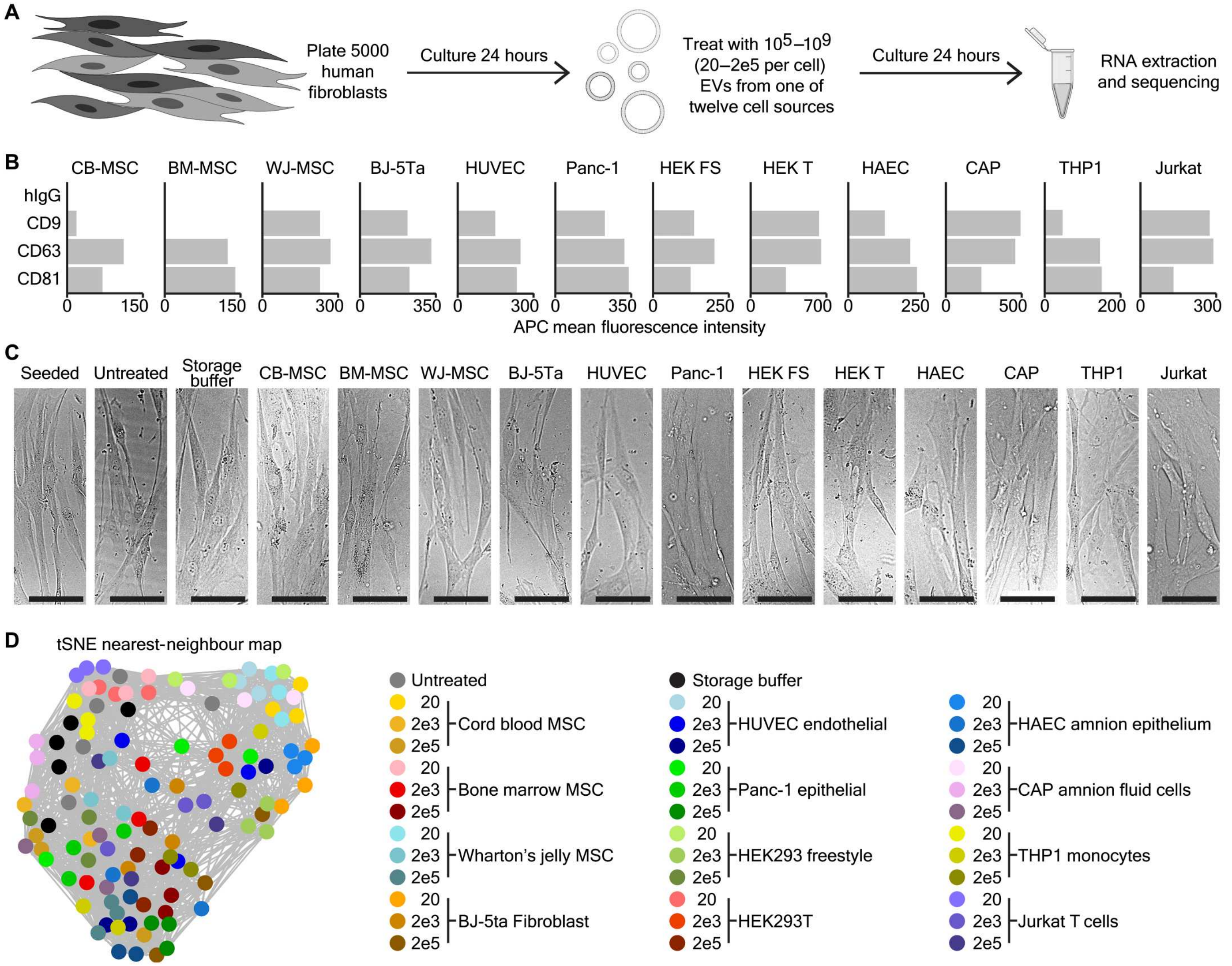

Fig. 1. RNA sequencing of fibroblasts treated with 12 EV types at three doses. (**A**) Schematic depicting the experimental design of EV treatment and RNA sequencing. (**B**) Multiplex bead–based flow cytometry analysis of control [immunoglobulin G (IgG)] and common EV tetraspanin markers (CD9, CD63, and CD81) capture beads using a pan-tetraspanin antibody mix for detection. (**C**) Bright-field images showing representative fibroblasts after seeding and untreated, or treated with storage buffer or 20,000 EVs derived from one of 12 different cell types, after 24 hours in culture. Scale bars, 100 μm. (**D**) tSNE-NN map of the fibroblast RNA sequencing data. This includes five untreated and five storage buffer–treated samples as controls, as well as triplicates from all EV treatment experiments at doses of 20, 2000, or 200,000 particles per cell.

with, Infomap clustering was performed to separate samples into four distinct clusters (#1 to 4; Fig. 2, A to C). The non–EV-treated samples were highly enriched within cluster 1, and thus these were used together as controls in future comparisons (Fig. 2, A, C, and D) (*29*). When assessing the cell source groupings, no clear enrichment of the samples in specific clusters was observed based on this parameter (Fig. 2, B and D). In contrast, a clear gradient of cells treated with different doses of EVs was observable, and these were enriched in specific clusters. Hence, cells treated with 20 EVs were mostly found in cluster 2, those exposed to 2000 EVs were concentrated in cluster 3, and samples incubated with 200,000 EVs per cell were primarily located in cluster 4 (Fig. 2, C and D).

To determine what genes separated fibroblasts treated with high and low doses of EVs, we performed differential expression analysis between controls and all samples treated with the same dose of EVs, regardless of their cell of origin (*30*). To understand the function of the genes that were differentially expressed, gene ontology analysis was applied to these sets of differentially expressed genes (*29*, *31*). This revealed that treatment with 200,000 EVs per cell significantly increased the expression of genes involved in autophagy, response to stress and transmembrane transport (Fig. 2E and fig. S2, A to C). In contrast, high doses of EVs repressed genes involved in exocytosis, extracellular matrix organization, and differentiation (Fig. 2E and fig. S2, A, D, and E). To confirm that the transcriptomic changes observed had functional consequences in EV treated cells, we made use of an established HEK293T line expressing CD63-ThermoLuc, which allows the EVs secreted from these cells to be quantified (Fig. 2F) (*32*). As suggested by our transcriptomic data, addition of increasing quantities of unlabeled exogenous EVs

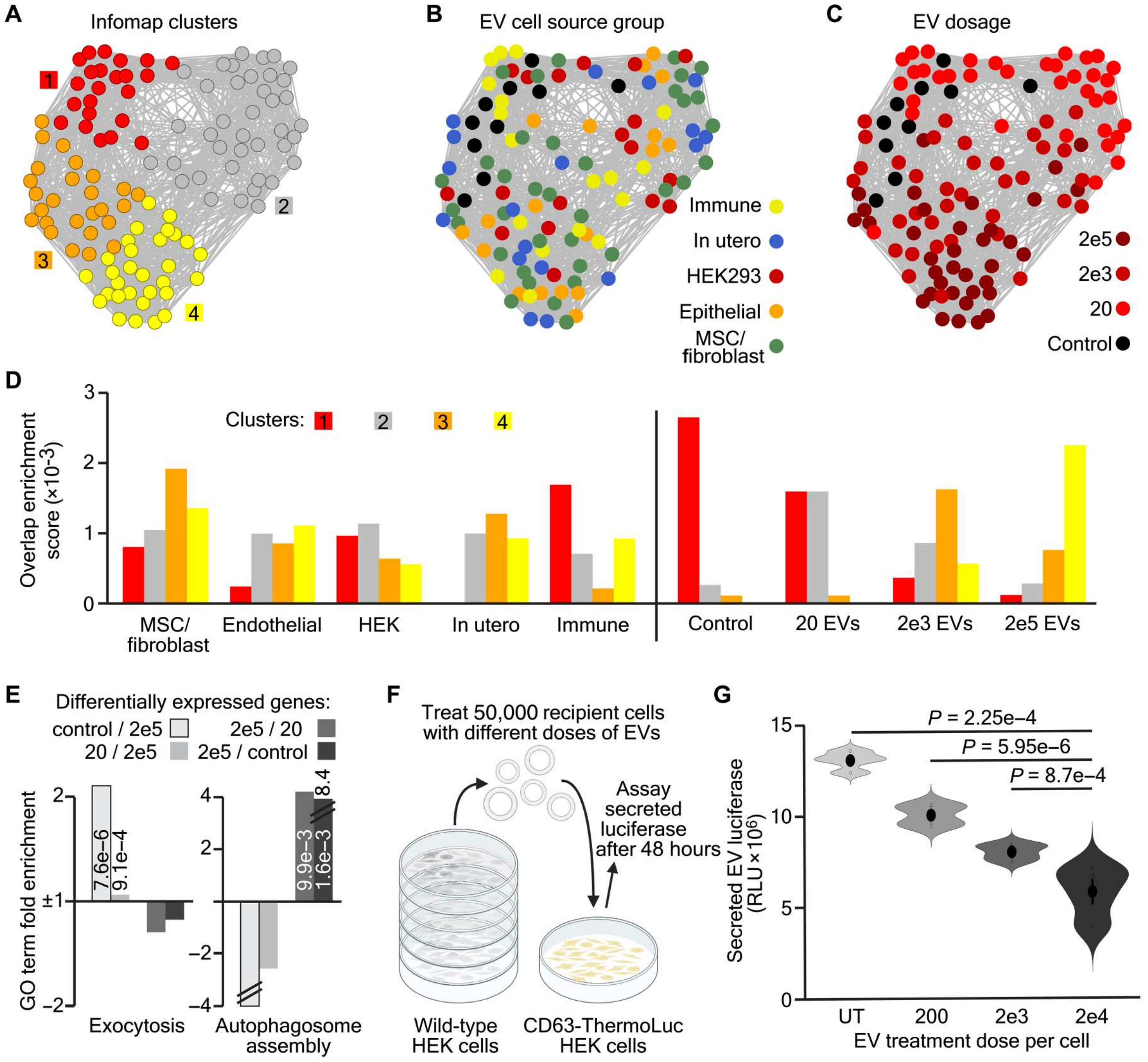

Fig. 2. EV treatment dose determines fibroblast transcriptomic responses. (**A** and **C**) tSNE-NN map from Fig. 1C with samples colored by Infomap cluster identity (A), EV cell type group (B), or EV dose per cell (C) fibroblast samples were treated with. (**D**) Overlap enrichment scores between samples' Infomap cluster identity and EV cell type group (left) or treatment dose (right). (**E**) Gene ontology term fold enrichment for genes up-regulated in control over cells treated with 200,000 EVs (white), 20 over 200,000 EV-treated cells (light gray), 200,000 over 20 EV-treated cells (dark gray), or 200,000 EV treated over control cells (darkest gray). *P* values for statistically significant terms are inset and negative values without fold change listed show no enrichment. (**F**) Schematic depicting the experimental design of exocytosis assays. (**G**) Violin plots of luciferase signals from CD63:ThermoLuc HEK cell EVs in media of untreated cells or those treated with 200, 2000, or 20,000 wild-type HEK EVs per cell ($n = 4$). Sample mean is shown as a large solid dot, SE as a horizontal line, and individual data points as rings, with statistics performed as two-tailed, unpaired *t* tests and *P* values displayed.

to the cell media resulted in a dosage-dependent decrease in the quantity of engineered EVs secreted by the cells (Fig. 2G).

To assess the cellular responses to EVs from the different cell sources, separate differential expression analyses were performed between controls and cells treated with EVs from each of the cell sources at each of the different doses. Unexpectedly, this revealed the most differentially expressed genes when comparing control cells to those treated with 20 EVs per cell, and the least differentially expressed genes in the cells exposed to 200,000 EVs per cell (Fig. 3A). To ensure that this was not due to structural differences in the different datasets, we checked the variation, fold change, and expression levels of the differentially expressed genes but found no statistical differences between these parameters at the different treatment doses (fig. S3, A to C). To understand whether the gene dysregulation observed represented a generalized cellular response to EVs, the overlap between the genes differentially expressed by the different cell sources was assessed at each EV dose. This revealed that the genes dysregulated by treatment with 200,000 EVs per cell were very similar, regardless of the EV cell source, while those dysregulated by 20 EVs per cell were specific for each cell source (Fig. 3B). This trend was maintained when comparing between doses, such that genes dysregulated by treatment with 200,000 EVs per cell were more similar to those altered by treatment with 2000 EVs per cell than with 20 EVs per cell (fig. S3D). This pattern further explains the distribution of samples observed in Fig. 1D; hence, global analysis disregards genes dysregulated in

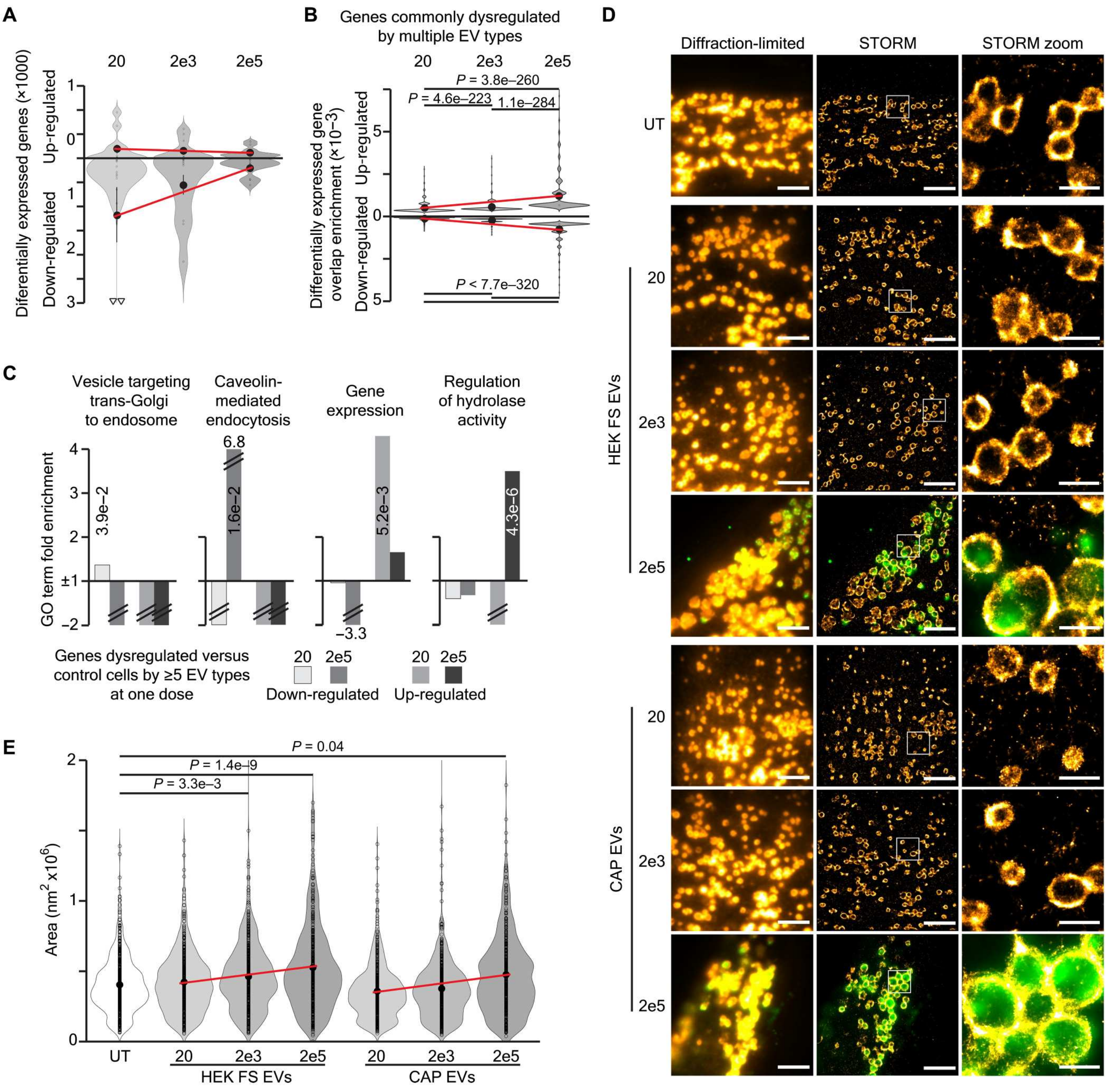

Fig. 3. Low doses of EVs produce unique transcriptional responses, while high doses induce lysosomal activity. (**A**) Violin plots of the raw number of significantly up- and down-regulated genes between control fibroblasts and those treated with each EV type at doses of 20, 2000, or 200,000 EVs per cell. Sample mean is shown as a large solid dot, SE as a horizontal line, and individual data points as rings. (**B**) Overlap enrichment scores of genes up- and down-regulated by the different EV types at doses of 20, 2000, or 200,000 EVs per cell. (**C**) Gene ontology term fold enrichment for genes up- or down-regulated by ≥5 EV types when treated with 20 (white and light gray) or 200,000 (dark gray and darkest gray) EVs per cell. *P* values for statistically significant terms are inset in bars. Values beside bars with hash marks show their fold enrichment beyond the graph scale, while negative bars with hash marks and no value represent no enrichment. (**D**) Representative microscopy images of LAMP1 immunostaining in HEK and CAP EV–treated fibroblasts at 24 hours. The diffraction-limited image of each spot is depicted, followed by the STORM image of the corresponding area and a zoom-in to the area indicated with the white rectangle. LAMP1 is shown in orange and green fluorescent protein (GFP)–EVs (only visualized in diffraction-limited mode) in green. Scale bars, 5 μm in diffraction-limited and full-view STORM images and 1 μm in STORM zoom-in images. (**E**) Violin plots of the LAMP1-labeled lysosomal area, as measured by STORM microscopy, in untreated fibroblasts ($n = 6$), or those treated with HEK or CAP ($n = 3$) cell–derived EVs at doses of 20, 2000, or 200,000 per cell. Statistics were performed by Wilcoxon signed-rank tests with Bonferroni correction for multiple comparisons, with significant values above control shown in (E).

individual samples. This result suggests that treatment with low doses of EVs produces profound, EV cell source–specific, transcriptional changes, while exposure to high doses of EVs produces a standardized cellular response.

To understand the biological processes commonly affected by EVs from multiple cell sources, gene ontology analysis was used to profile genes that were up- or down-regulated by at least five types of EVs at a single dose (table S1). This showed that genes down-regulated by treatment with 20 EVs per cell involved anterograde vesicle targeting, while those that were up-regulated participated in gene expression. In contrast, genes commonly down-regulated by treatment with 200,000 EVs per cell were involved in endocytosis, while those that were up-regulated had roles in lysosomal hydrolase activity (Fig. 3C). To functionally confirm that high doses of EVs up-regulate lysosomal activity, we turned to high-resolution stochastic optical reconstruction microscopy (STORM) to visualize lysosomal dynamics by Lysosomal-Associated Membrane Protein 1 (LAMP1) staining (*33*, *34*). Hence, exposing fibroblasts to CD63-mNeonGreen (mNG)–engineered EVs from HEK293 freestyle or CAP EVs revealed that lysosomes appeared larger in cells treated with high doses of EVs. Moreover, these enlarged lysosomes were associated with abundant CD63-mNG EVs (Fig. 3, D and E). Last, quantifying the lysosomal area using a machine learning–aided workflow confirmed that lysosomes were significantly larger when cells were exposed to the 200,000 EVs per cell in comparison to the 20 EV per cell dose (Fig. 3D and fig. S4, A to C).

This functional in vitro confirmation of our transcriptomics data led us to question whether the same responses to high EV doses could be observed in vivo. To judge this, we injected mice with HEK freestyle EVs, which were engineered to express CD63-mNG, dissected their livers 3 hours after injection and sorted 192 mNG^+ cells for single-cell RNA sequencing (Fig. 4, A and B, and fig. S5A). Following mapping to the mouse genome and quality control cell filtering, we produced a tSNE-NN map of the remaining cells based on the most variable genes in the dataset (fig. S5, B to D). The map showed no obvious groupings, and labeling with common markers of the most abundant cell types in the liver (hepatocytes: *ASGR1* and *A1BG*; Kupffer cells: *CD14* and *VSIG4*; and endothelial cells: *EDNRB* and *OSMR*) suggested the cells to be hepatocytes (Fig. 4C and fig. S5E) (*28*, *35*). To separate these cells based on the dose of EVs they were exposed to, we mapped the reads from each cell to the human genome and assessed the ratio of human to mouse reads in each cell (Fig. 4D). Since HEK cells are a human cell line, this allowed us to separate the single cells into four groups describing their EV dose (low, medium low, medium high, and high). By comparing the distribution of these groups with unbiased clustering, we observed that low– and high–EV-dosed cells were enriched in separate clusters (Fig. 4, E to G). Differential expression analysis between low– and high–EV-dosed cells revealed high–EV-dosed cells to be enriched in genes involved in lysosome acidification, assembly, and localization. In contrast, low–EV-dosed cells showed higher expression of genes involved in EV biogenesis, endocytosis, and homeostasis (Fig. 4H).

Next, we sought to directly assess differences in the cellular response to EVs from the various cell sources. Since the most robust and unique transcriptional responses to EVs were observed at the lowest doses, control fibroblast transcriptomes were mapped together with those exposed to 20 EVs per cell from the different cell sources (Fig. 5A and fig. S6A). Quantifying the clusters where different EV-type treated samples were distributed suggested that immune- and HEK-derived EVs produced the least, while in utero- and MSC-derived EVs yielded the greatest, transcriptional changes in comparison to control cells (Fig. 5B). To study the genes regulated by EVs from specific cell types, we analyzed those significantly up- or down-regulated by each EV type in at least two of the dosage experiments performed (Fig. 5C and table S2). In general, the genes regulated by MSC- and epithelial cell–derived EVs were relatively similar to one another, while HEK-, in utero–, and immune cell–derived EVs elicited distinct cellular responses. When comparing the effects of EVs originating from cells of the same group, only epithelial cell–derived EVs regulated highly overlapping gene sets (fig. S6B).

One possibility is that different types of EVs induce responses that reflect the cellular or niche activities of their source cells. To test this hypothesis, gene ontology analysis was applied to the genes identified as regulated by each EV type (Fig. 5D). Wound healing is often associated with MSC-derived EVs (*24*), and this analysis revealed that EVs from all of the MSC, as well as HAEC and THP1, lines induced genes involved in this process. Similarly, homotypic cell adhesion is an important property of epithelial cells, and the EVs from both of these cell types, in addition to those from Wharton's jelly connective mucosa and CAP cells, promoted the expression of such genes. In contrast, only HEK cell–derived EVs up-regulated genes involved in amino acid activation, only in utero cell–derived EVs induced genes promoting DNA synthesis, and only immune cell–derived EVs elicited a cellular response involving immune system processes (Fig. 5D). To judge whether these transcriptional effects were translated into functional cellular activities, we assessed the effect of EVs from different cell types on the angiogenic capacity and proliferation of HUVEC cells as quantifiable proxies of wound healing and DNA synthesis, respectively. Thus, we first confirmed that these phenotypes were robustly reflected in the gene ontology analysis of MSCs and in utero cell–derived EV treated cells, respectively (fig. S6, C and D). We then applied cord blood MSC–, HEK293T-, CAP-, and THP1 cell–derived EVs to HUVEC endothelial cells in a tube formation assay. These results showed only cord blood MSC EVs to significantly stimulate endothelial cell branching, in agreement with our transcriptional data (Fig. 5, E and F). Next, we assessed the proliferation of the treated cells and found that CAP-, and to a lesser extent, HEK-, and MSC cell–derived EVs were able to activate the cell cycle (Fig. 5G). These results confirmed that the EV-specific transcriptional patterns detected in the sequencing data were borne out in functional cellular activities.

To further break down the cellular response to individual EV types, we began by mapping control fibroblasts with those exposed to different doses of each of the EV types separately. This showed the 20 EV per cell dose to be most separated from control cells as frequently as the 200,000 EV per cell dose, and that these two doses most often clustered away from each other with control cells intervening (fig. S7). To characterize the genes that responded to individual EV types, the genes dysregulated by EVs from the same cell source group were compared (table S2). In the MSC/fibroblast group, the transcriptional changes observed were most similar in response to cord blood MSC– and BJ-5ta–derived EVs (fig. S8A,B). The cellular responses to different types of MSC-derived EVs were appropriate to their tissue of origin, such that bone

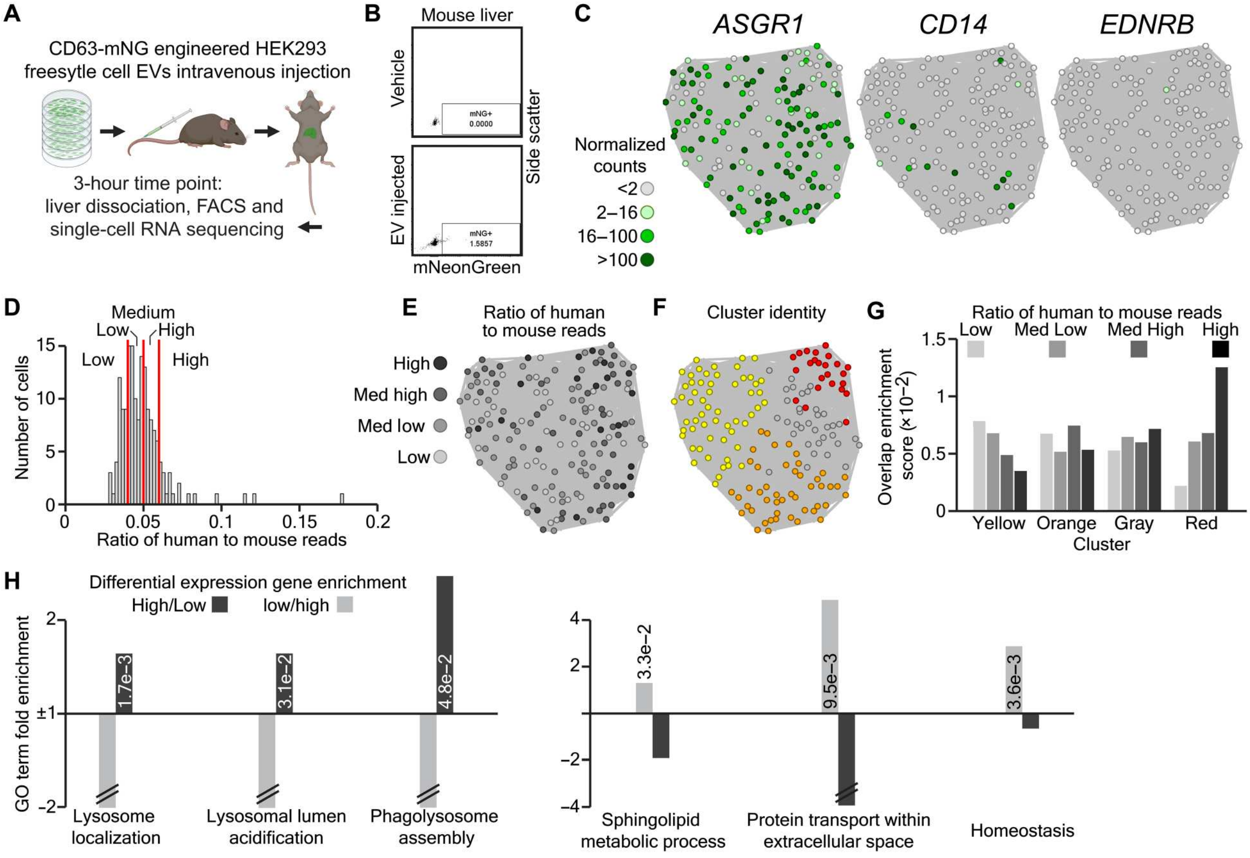

Fig. 4. High doses of EVs activate lysosomal and repress membrane trafficking genes in vivo. (**A**) Schematic depicting CD63-mNG HEK293 freestyle EV injection, liver dissection, and single-cell RNA-sequencing experiment. (**B**) FACS dot plots and gating of single-cell mNeonGreen levels generated during cell sorting of control and EV-injected mouse liver ($n = 2$). (**C**) Single-cell RNA-sequencing tSNE-NN maps colored on the basis of the expression of a hepatocyte (*ASGR1*), Kuppfer (*CD14*), or endothelial cell (*EDNRB*) marker. (**D**) Histogram showing cell's ratios of human to mouse mapped reads and the cutoffs (red lines) used to separate populations of cells having taken up low, medium low, medium high, and high numbers of EVs. (**E** and **F**) tSNE-NN maps colored on the basis of cell's human to mouse mapped reads group (E) or Infomap cluster identity (F). (**G**) Overlap enrichment scores between cells' Infomap cluster identity and human to mouse mapped reads group. (**H**) Gene ontology term fold enrichment for genes up-regulated in high (dark gray) or low (light gray) human to mouse mapped reads cells.

marrow MSC–derived EVs regulated immune processes and Wharton's jelly MSC–derived EVs up-regulated DNA replication (fig. S8C). Similarly, both epithelial cell EVs up-regulated aerobic respiration genes and down-regulated those associated with epithelial migration (fig. S9, A and B). Unexpectedly, EVs from adherent and suspension HEK cells produced relatively unique responses, with suspension cell EVs stimulating proliferation and adherent cell EVs activating metabolism genes (fig. S10, A and B). The genes regulated by both in utero–derived EV types were involved in cell cycle activity, a trait that was also observed in response to EVs derived from other cells supporting fetal growth (figs. S8C, S9C, and S11, A and B). Last, immune cell EVs also produced responses that mirrored the cell types they were derived from. For instance, THP-1 monocyte EVs induced production of interleukin-8 (IL-8), while Jurkat T cell EVs up-regulated genes involved in T cell proliferation and both stimulated a response to the cytokine IL-3, which is secreted by cells of both the myeloid and lymphoid lineages (fig. S12, A and B) (*36*).

As one of the greatest interests in EV research is in using them to deliver therapeutics, one important question is whether EVs from certain cell sources are more readily taken up than others by target cells. To assess this, we first performed RNA sequencing directly on the different EV samples to capture the mRNAs that each carried, which appeared to be long (fig. S13A). To visualize the differences in mRNA cargo between EVs from different cell sources, the EV transcriptomes were mapped together and clustering was performed on this dataset (Fig. 6A and fig. S13B). Except for the in utero–derived EVs, the associations between EV transcriptomes from the different cell groups resembled those of their cellular transcriptional responses, with HEK and epithelial cell EVs separating MSC and immune cell–derived samples (Figs. 5B and 6B). Next, we identified transcripts that were consistently enriched or depleted in each EV type by performing differential expression analysis between them (Fig. 6C). Comparing these genes showed that EVs from the same cell source group carried relatively overlapping transcripts, except in the case of HEK cells cultured under different conditions (Fig. 6C and fig. S13C).

To track the relative cellular association of EVs at different doses, the presence of transcripts that were most abundant in each type of EVs, but not expressed in control fibroblasts, was assessed. As expected, this showed that cells treated with the lowest doses of EVs tended to contain the fewest EV-derived transcripts. However, most

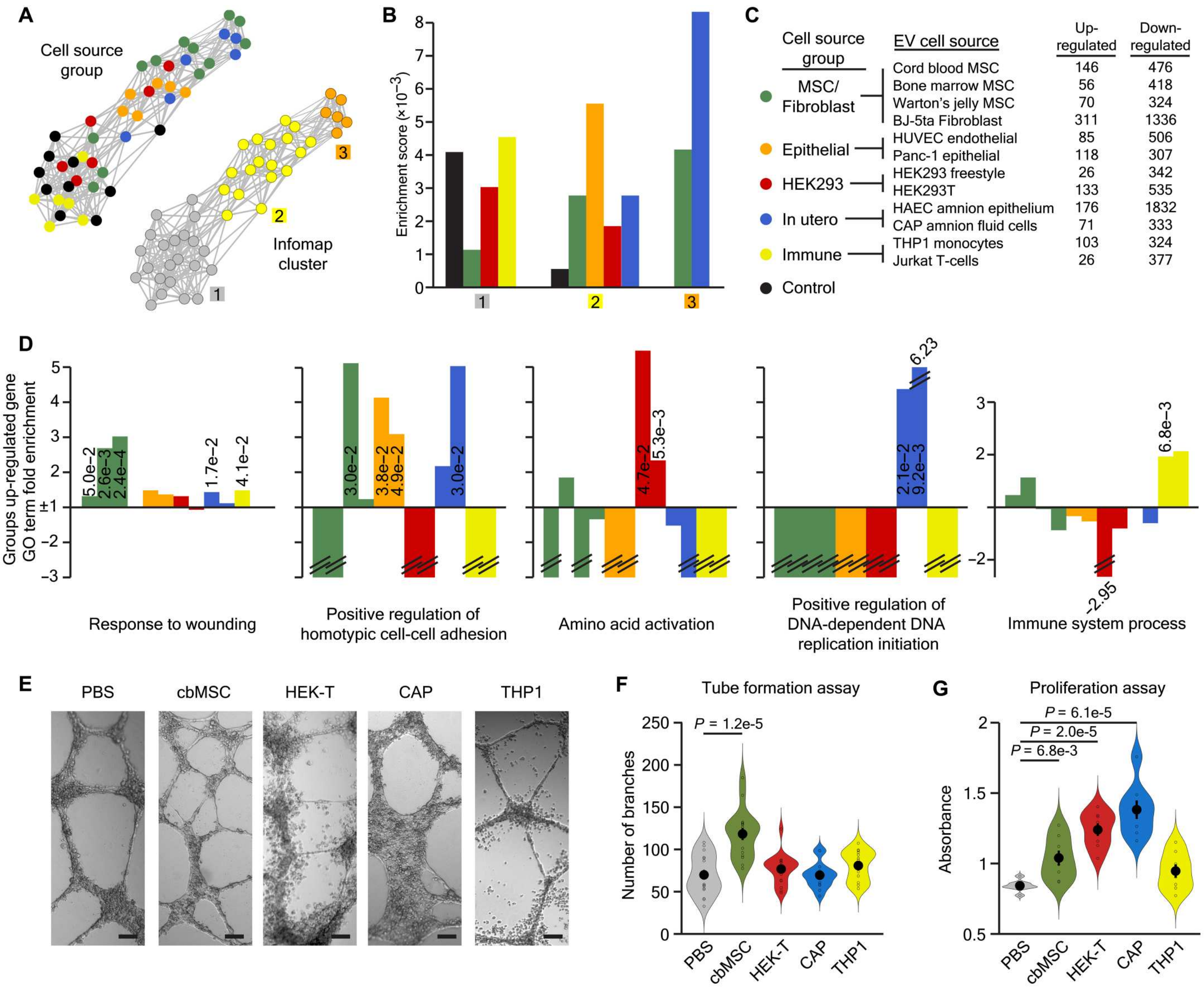

Fig. 5. EVs produce transcriptional responses reflective of their cell source. (**A**) tSNE-NN map of control and 20 EV per cell treated fibroblast transcriptomes colored by the cell source group of the EVs they were treated with or by their Infomap cluster. (**B**) Overlap enrichment scores between samples' Infomap cluster identity and the EV cell type group they were treated with. (**C**) Numbers of genes significantly up- or down-regulated by each type of EV at more than one dose and their cell source group. (**D**) Gene ontology term fold enrichment for genes robustly up-regulated in fibroblasts by each of the EV types, colored on the basis of their cell source group and ordered according to (C). *P* values for statistically significant terms are inset and negative values without fold change listed show no enrichment. (**E**) Bright-field images of HUVEC cells during the invasion assay. Scale bars, 100 μm. (**F**) Quantification of the number of branches formed by HUVEC cells exposed to EVs from different cell sources ($n = 14$ to 17). (**G**) Quantification of cell proliferation in HUVEC cells exposed the EVs from different cell sources ($n = 8$). Sample mean is shown as a large solid dot, SE as a horizontal line, and individual data points as rings, with statistics performed as two-tailed, unpaired *t* tests.

EV types did not increase the association of their transcripts with target cells at higher doses, as eight of 12 EV types showed little change or even lower levels at higher doses. The four EV types that exhibited a dosage-dependent increase in their transcripts above control cell levels were derived from Panc-1, HAEC, THP1, and Jurkat cells (Fig. 6D). This pattern of association also held true when unrelated transcripts, previously found to be specifically enriched in each EV type, were analyzed (Fig. 6C and fig. S13D).

DISCUSSION

In recent years, research on the potential medical applications of EVs, in diagnostics and therapeutics, have raced ahead of our understanding of their basic biological functions. This is illustrated by the experimental design of most EV research, which applies one type of EV at a single dose (*14*). However, by performing a global analysis of the effects of EVs from 12 cell sources at three different doses, our findings have revealed fundamental pitfalls in this approach. Hence, our results demonstrate that dosing should be a fundamental consideration when testing EVs as therapeutic modalities

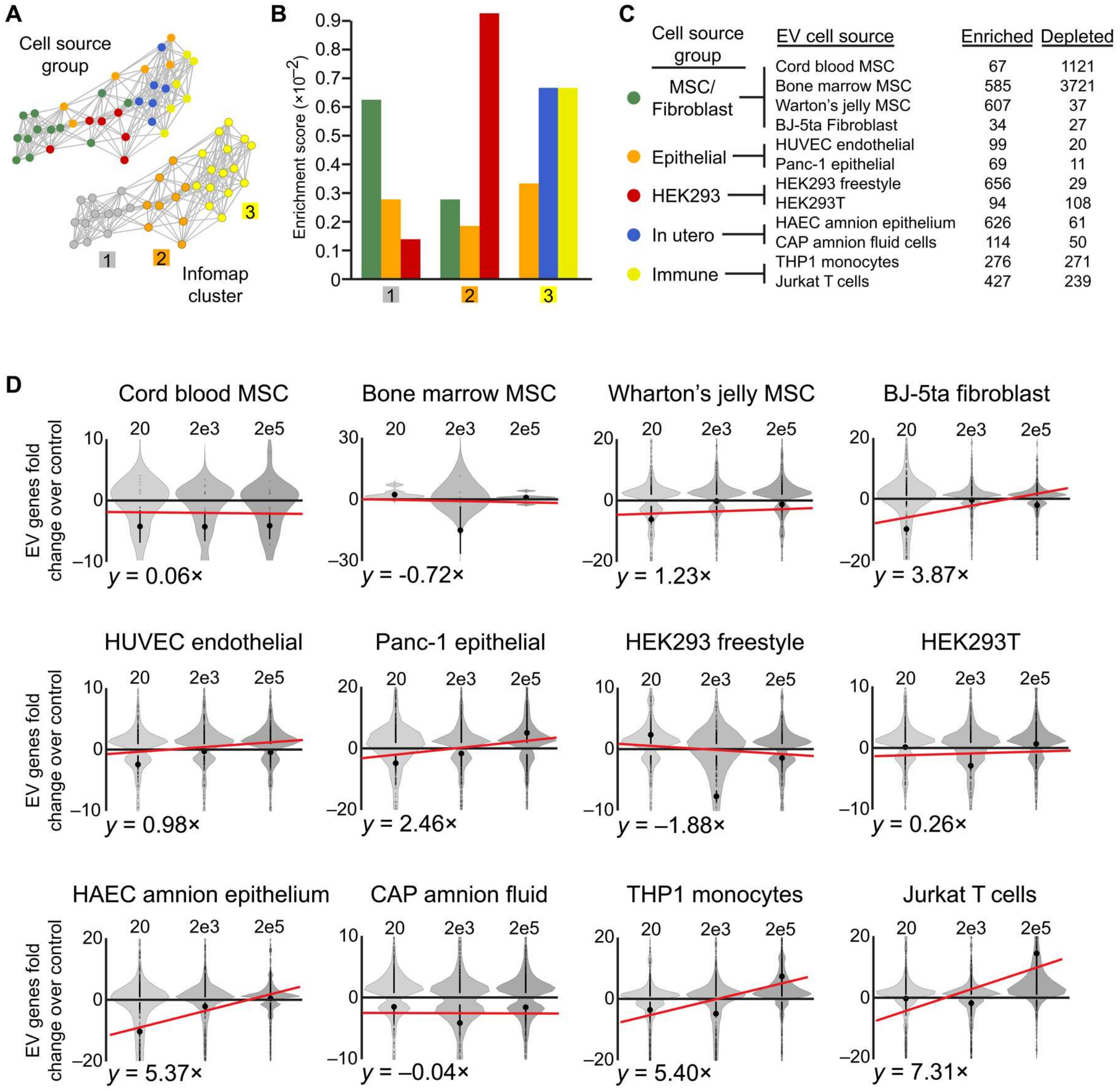

Fig. 6. EV-derived transcripts from different source cells vary in their associations with recipient cells. (**A**) tSNE-NN map of EV transcriptomes colored by their cell source group or Infomap cluster. (**B**) Overlap enrichment scores between EV's Infomap clusters and cell source group. (**C**) Numbers of genes repeatedly enriched or depleted in each EV type when compared with at least three other EV types. (**D**) Violin plots of the fold difference in fibroblast expression of transcripts highly abundant in each type of EV over their basal levels in control fibroblasts, when treated with 20, 2000, or 200,000 EVs per cell. Sample mean is shown as a large solid dot, SE as a horizontal line, and individual data points as rings. Best fit line is overlaid in red, with the slope of each line displayed below.

and that performing dose-response experiments should be a prerequisite to publication.

There are several important considerations to the interpretation of this work. First, although the doses analyzed were chosen on the basis of those used in literature, the highest doses are unlikely to reflect physiological conditions and likely overload the endocytic machinery. Second, isolation of EVs using tangential flow filtration (TFF) retains large proteins, which may be counted as EVs and affect dosing or induce non–EV-mediated signaling. Last, despite using an optimized storage buffer (*22*), the freezing of EVs after isolation may denature proteins and contribute to the stress responses we observed. Despite these caveats, we found that the greatest transcriptional responses were observed when EVs were applied at low doses. In contrast, high doses activated genes involved in lysosomal activity and increased their size, regardless of the EV source. Although the 3-hour time point studied here in vivo does not reflect the rapid uptake of nanoparticles by Kupffer cells in the liver, it allowed us to confirm the vesicle trafficking transcriptional phenotypes we observed in vitro and demonstrate that dosing will also have an impact on the clinical application of EVs. Thus, it was interesting that EV-borne transcripts were only detected in target cells after treatment at high doses. Together, these results suggest that EV-mediated surface protein signaling is most robust at low doses, as this likely best reflects the situation in vivo, while the detection of specific cargo requires higher doses of EVs. However, such high doses are likely only necessary because of the low

efficiency of most EV engineering strategies. This suggests that optimizing the loading and targeting methods could reduce the dose of EVs necessary and improve their therapeutic efficacy. At the very least, standardized and comparable dosing should become common practice in EV research.

Another significant finding of this work is that EVs from different cell sources produce specific transcriptional responses in recipient fibroblasts. These cellular responses corresponded with the activities and phenotypes of the EV source cells. Hence, MSC-derived EVs induced wound healing, epithelial cell–derived EVs up-regulated cell adhesion genes, in utero–derived EVs promoted proliferation, and immune cell–derived EVs activated genes involved in immune system processes. However, these effects are dependent on numerous variables during the in vitro production of EVs, as adherent and suspension HEK cultures produced very different EVs. Mechanistically, both EV surface proteins and internal cargo have both been shown to play a role in influencing cellular responses. Here, the inverse correlation between doses showing EV-transcript delivery and transcriptional changes suggests that EV cargo has a less potent effect in this regard and indicates that therapeutics are best targeted to the EV surface. Nonetheless, most EV pharmaceuticals are designed as cargoes, and in this respect, it was concerning that most EVs did not deliver their transcripts in a dose-dependent fashion. Although it is possible that different EV types bear varying quantities of mRNA, it was thus interesting that the two EV types most capable of this were derived from immune cells. As these cell types also produced among the most muted transcriptional changes in recipient cells, these may represent a promising tool for the delivery of therapeutic molecules.

The field of EV research has grown rapidly based on the promise that they hold for the unprecedented targeting of therapies. Although this has led to a proliferation of engineering strategies and disease targets, our basic knowledge of how cells react to endocytosed EVs is lacking. This work provides important insights into the target cell response to EVs derived from different cell types at different doses. Although our global approach is different to the assessment of a targeted therapeutic, this research demonstrates that the cells used to produce EVs and the doses applied should be carefully assessed to fit each project's specific goals.

MATERIALS AND METHODS

EV source cell culture

Cell lines were cultured in the following media: Immortalized, human bone marrow–, and umbilical cord–derived mesenchymal stromal cells (hTert + MSCs) were cultured in MEM-α modification medium (containing L-glutamine; Thermo Fisher Scientific) supplemented with basic fibroblast growth factor (5 ng/ml; Sigma-Aldrich, F0291). Wharton's jelly MSCs were cultured in Dulbecco's modified Eagle's medium (DMEM)–low glucose [containing GlutaMAX-I and sodium pyruvate; glucose (1 g/liter); Invitrogen], BJ-5ta fibroblast cells (fibroblast immortalized with TERT) were cultured with 4:1 mixture of Dulbecco's medium [containing 4 mM L-glutamine, glucose (4.5 g/liter), and sodium bicarbonate (1.5 g/liter)] and Medium 199 [Hygromycin B (0.01 mg/ml)/10687010; Thermo Fisher Scientific], HUVEC were cultured in Vascular Cell Basal Medium (PCS-100-030, ATCC), supplemented with endothelial cell growth Kit-VEGF [PCS-110-041, American Type Culture Collection (ATCC)], PANC-1 cells were cultured in DMEM/F12 (containing 2.5 mM L-glutamine and 15 mM Hepes], HEK293T cells were cultured in DMEM [containing GlutaMAX-I and sodium pyruvate; glucose (4.5 g/liter); Invitrogen], HEK293 Freestyle suspension cells (HEK293FS; Thermo Fisher Scientific) were cultured in FreeStyle 293 Expression Medium (Thermo Fisher Scientific), and CAP amnionic fluid cells were cultured in serum-free protein expression media (Thermo Fischer Scientific) with GlutaMAX-I and puromycin (2 μg/μl), in 125-ml polycarbonate Erlenmeyer flasks (Corning) in a shaking incubator (Infors HT Minitron) according to the manufacturer's instructions. HUAC amniotic epithelium, which were isolated from human placentae as previously described (*37*), were cultured in Dulbecco's medium [containing 4 mM L-glutamine, glucose (4.5 g/liter), and sodium bicarbonate (1.5 g/liter)] supplemented with nonessential amino acid, β-mercaptoethanol, and 5% Stemulate (Cook Regentec). THP1 monocyte and Jurkat T cell lines were cultured in RPMI-1640 medium (containing GlutaMAX-I and 25 mM Hepes; Invitrogen). Unless indicated otherwise, all cells were supplemented with 10% fetal bovine serum (FBS; Invitrogen) and 1× antibiotic-antimycotic (anti-anti) (Thermo Fisher Scientific). All cell lines were grown at 37°C and 5% CO_2 in a humidified atmosphere and regularly tested for the presence of mycoplasma.

EV production and purification

For EV harvesting, cell culture–derived conditioned media (CM) was changed to OptiMem (Invitrogen) 48 hours before harvest of CM as described before (*38*). Unless indicated otherwise, all CM samples were directly subjected to a low-speed centrifugation step at 500*g* for 5 min followed by a 2000*g* spin for 10 min to remove larger particles and cell debris. Precleared cell culture supernatant was subsequently filtered through 0.22-μm bottle top vacuum filters (Corning, cellulose acetate, low protein binding) to remove any larger particles. EVs were prepared by TFF. For the TFF EV preparation, precleared CM was concentrated via TFF by using the KR2i TFF system (Spectrum Labs) equipped with modified polyethersulfone hollow fiber filters with 300-kDa membrane pore size (MidiKros, 370 cm^2 surface area, Spectrum Labs) at a flow rate of 100 ml/min (transmembrane pressure at 3.0 psi and shear rate at 3700 sec^{-1}) as described previously (*38*). Amicon Ultra-0.5 10-kDa MWCO spin-filters (Millipore) were used to concentrate the sample to a final volume of 100 μl. EV samples were stored at −80°C in phosphate buffered saline (PBS)– human albumin trehalose (HAT) buffer (*22*).

Nanoparticle tracking analysis

Nanoparticle tracking analysis (NTA) (*39*) was applied to determine particle size and concentration of all samples using the NanoSight NS500 instrument equipped with NTA 2.3 analytical software and an additional 488-nm laser. The samples were diluted in 0.22-μm filtered PBS to an appropriate concentration before being analyzed. At least five 30-s videos were recorded per sample in light scatter mode with a camera levels of 11 to 13. Software settings for analysis of scatter particles were kept constant for all measurements (screen gain, 10; detection threshold, 7). The analysis was performed with the screen gain at 10 and detection threshold at 7 for all measurements.

EV uptake experiments
Five thousand human primary fibroblasts (NIGMS Human Genetic Cell Repository #GM08402) were seeded into flat bottom 96-well plates and incubated with 90 μl of full medium with 10 μl of PBS-HAT buffer containing 1×10^5, 1×10^6, 1×10^7, 1×10^8, or 1×10^9 EVs for 24 hours. Cells were then washed twice with PBS and analyzed.

RNA sequencing
Cell or EV RNA was extracted (*32*) and precipitated as previously described (*3*) by incubating 500 μl of TRI reagent (Sigma-Aldrich), adding 100 μl of chloroform and shaking vigorously. After a 15-min incubation, samples were centrifuged at 12,000*g* for 15 min at 4°C and 300 μl of aqueous phase was mixed with 300 μl of isopropanol, 30 μl of 3 M sodium acetate, and 1 μl of pellet paint (Merck) and incubated over night at −20°C. The next morning, samples were centrifuged at 20,000*g* for 30 min at 4°C, the pellets were washed two times with 700 μl of 70% ethanol, before drying and resuspending in 15 μl of elution buffer (Qiagen). RNA concentrations were measured using Qubit RNA high-sensitivity assay (Thermo Fisher Scientific) and 2 ng was used to generate full-length complementary DNA by Smart-seq2, which uses an oligo dT primer (*23*). Fifty–base pair single-end reads were sequenced on a HiSeq3000 (Illumina), converted to fastq using bcl2fastq, adapters trimmed using Trim Galore, and the resulting reads aligned to the ENSEMBL human transcriptome GRCh37 or ENSEMBL mouse transcriptome GRCm39 using Tophat 2.1.1. To generate the normalized count matrix, DEseqDataSetFromMatrix and estimateSizeFactors were applied from the DEseq2 package in R (*30*).

Clustering, differential expression, and gene ontology and overlap enrichment analysis
Variable genes above six normalized counts and transcriptome mapping was performed as previously described (*28*, *29*). Briefly, Euclidian distances between samples were derived from tSNE analysis using the Rtsne package and maps were assembled by applying force-directed connections between each sample and its 25 NNs for Figs. 1 and 2 and 40 NNs for Fig. 4. Infomap clustering and visualizations were produced using the igraph package in R. Differential expression between groups indicated was performed using the Deseq2 package in R. Genes with an adjusted *P* value below 0.05 were separated on the basis of whether they were up-regulated or down-regulated and analyzed using panther.org complete biological processes statistical overrepresentation test version 10.5281/zenodo.4081749, released 09 October 2020. A control gene set was constructed from all up- and down-regulated genes graphed together and the fold enrichments displayed for each individual group were calculating by dividing their enrichment by that of the same term in the control group. Gene overlap enrichment was performed as: (# overlapping genes) / (# genes in group one) × (# genes in group two) (*29*).

Relative gene similarity and heatmaps
The relative similarity between two samples within a group was calculated by taking ratio of the number of significantly differentially expressed genes between two groups over the average number for each individual sample. This produced enrichment scores that were then averaged to give the similarity between groups, whereby relatively low numbers of differentially expressed genes between two groups produced high similarity scores. Heatmaps were then generated using the gplots package in R.

Exocytosis quantification
Cellular exocytosis levels were judged as in (*32*) by seeding 50,000 HEK293T:CD63-ThermoLuc cells per well in 24-well plates and culturing in full medium for 48 hours. Media was then changed to OptiMEM with or without the number of wild-type HEK EVs indicated and cultured for a further 48 hours. Media was then collected, centrifuged at 2000*g* for 10 min, and lysed in 0.1% Triton-X. Luciferase signal was then used as a proxy for EV secretion from cells by injecting 25-μl Luciferase reagent and measuring on a GloMax96 Microplate Luminometer (Promega).

STORM microscopy and lysosome size quantification
Fibroblasts were fixed for 15 min in 4% paraformaldehyde (PFA) and 0.2% glutaraldehyde, washed three times in PBS, and stored at 4°C until imaged. The immunostaining was conducted right before the STORM image acquisition. Briefly, the samples were permeabilized with 0.05% Triton X-100 (Sigma-Aldrich) in PBS for 5 min at room temperature. A 1.5-hour blocking step in 3% bovine serum albumin (BSA; Thermo Fisher Scientific) in PBS was performed, followed by overnight +4°C incubation of the primary antibody (anti-LAMP1 D2D11 XP Rabbit mAB, Cell Signaling Technology, #9091), diluted 1:200 in 3% BSA. Following washes, the samples were treated for 1.5 hours with 1:1000 diluted secondary antibody (donkey anti-rabbit Alexa Fluor 647, Invitrogen, A31573). The antibodies were fixed with 2% PFA for 10 min at room temperature, and nuclei were stained with 4′,6-diamidino-2-phenylindole (DAPI; Invitrogen, D1306; 1:5000 dilution in PBS, 5 min at room temperature).

Imaging was performed as in (*30*). Briefly, samples were soaked in imaging buffer and STORM was performed on a Nikon Ti Eclipse inverted microscope. Green fluorescent protein (GFP) and DAPI were acquired in diffraction limited mode, while LAMP1 was imaged as STORM. A 256 × 256 pixels region of interest was imaged and a diffraction-limited image was acquired for reference before starting the STORM acquisition. Each image consists of 30,000 frames, with an exposure time of 30 ms per frame, and four to five images were acquired for each sample. Image stacks were reconstructed with the ThunderSTORM plugin in Fiji with drift correction and sigma-based filtering (*34*). The final pixel size in the STORM visualizations was 8.7 nm. An overlay of the STORM image of LAMP1 staining and diffraction-limited images of GFP-EVs and nuclei of each region of interest is presented.

Lysosomes in a subset of STORM images were manually annotated in Fiji and a mask image was created using the region of interest map function of the LOCI plugin. StarDist 2D deep learning model was trained for 80 epochs on 14 paired image patches of original STORM images and manually annotated images (*40*). The quality of the trained model was evaluated with four validation images not used for the training (manually annotated the same way as the training data). The areas of objects identified with the deep learning model were determined with CellProfiler (version 3.1.9.). The different groups had the following number of objects (lysosomes): UT, 772; HEK 20 EVs, 566; HEK 2000 EVs, 580; HEK 200,000 EVs, 558; CAP 20 EVs, 600; CAP 2000 EVs, 605; and CAP 200,000 EVs, 575.

Single-cell sorting, RNA sequencing, and bioinformatic analysis of mouse liver cells

All experiments were approved by the Swedish Ethical Review Authority of the Stockholm Region. EVs were generated and isolated from HEK293 freestyle:CD63-mNeonGreen cells as described above. EVs (5×10^{11}) were injected intravenously into C57Bl6 (n = 2) mice, and after 3 hours, livers were dissected and single cells dissociated by physical disruption and passing through a 70-μm cell strainer. A total of 192 single cells were then sorted on a FACSAria Fusion instrument (BD Biosciences) into lysis buffer (*23*) in 96-well plates by gating on single, DAPI-negative and mNeonGreen-positive cells. Sorted cells were processed for sequencing as described above. Reads were then mapped to the ENSEMBL mouse (GRCm39) and human genomes as described above. Quality control (QC) was performed on the basis of cells total mouse genome mapped reads (10,000 < passing cutoff < 300,000), genes detected above two normalized counts (500 < passing cutoff < 3000), and the ratio of mitochondrial genome mapped reads (passing cutoff < 0.05). The ratio of human to mouse genome mapped reads was calculated for all cells passing QC, and these were used to separate them into four groups (low < 0.04 < medium low < 0.05 < medium high < 0.06 < high), which we used as a proxy for the quantity of EVs a cell had been exposed to. Last, cells passing QC were mapped using the tSNE-NN, with cells linked to their 40 NNs, and differential expression, performed on random pseudobulk cell groups of five or six cells dependent on the total number of cells in a group, strategies described above.

Tube formation and proliferation assays

Tube formation assay was used to evaluate the angiogenic potential of EVs by coculturing with HUVECs on Matrigel (Corning, Belford, USA). For Matrigel tube formation assay, HUVECs were serum starved by culturing in epithelial cell basal medium-2 (ATCC) with 2% FBS for 5 hours. The serum starved cells were plated at the density of 4×10^4 cells per well on Matrigel, which coated the wells of 96-well plates and were equilibrated with EBM-2 medium (Lonza) (containing EVs with indicated concentrations). As a control, HUVECs were cultured alone and with PBS. When cultured on Matrigel, endothelial cells aligned themselves into a network structure within 12 hours and the results were documented photographically using an inverted microscope (Olympus) at 10× magnification and then the length of tube formation, node numbers were calculated using ImageJ Software (National Institutes of Health, Bethesda, Maryland). Proliferation was measured using a WST-1 assay according to the manufacturer's instructions (Sigma-Aldrich).

Quantification of EV uptake

We first defined EV gene sets either by their enrichment compared to the other groups (Fig. 5C) or abundance above an average of 100 normalized counts in each of the different EV types. We then subtracted any gene expressed above an average of one normalized count in the control fibroblasts from these gene sets. We then gave genes with 0 normalized counts a value of 0.01 and looked at the expression of these gene sets in the fibroblasts exposed to the different doses of the corresponding EVs. A linear best fit curve was calculated in Excel for each set and the slope of that curve is shown inset in each graph.

Supplementary Materials

This PDF file includes:
Figs. S1 to S13
Legends for tables S1 to S3

Other Supplementary Material for this manuscript includes the following:
Tables S1 to S3

REFERENCES AND NOTES

1. H. Valadi, K. Ekström, A. Bossios, M. Sjöstrand, J. J. Lee, J. O. Lötvall, Exosome-mediated transfer of mRNAs and microRNAs is a novel mechanism of genetic exchange between cells. *Nat. Cell Biol.* **9**, 654–659 (2007).
2. J. Skog, T. Würdinger, S. van Rijn, D. H. Meijer, L. Gainche, M. Sena-Esteves, W. T. Curry Jr., B. S. Carter, A. M. Krichevsky, X. O. Breakefield, Glioblastoma microvesicles transport RNA and proteins that promote tumour growth and provide diagnostic biomarkers. *Nat. Cell Biol.* **10**, 1470–1476 (2008).
3. D. W. Hagey, M. Kordes, A. Görgens, M. O. Mowoe, J. Z. Nordin, C. F. Moro, J.-M. Löhr, S. El Andaloussi, Extracellular vesicles are the primary source of blood-borne tumour-derived mutant KRAS DNA early in pancreatic cancer. *J. Extracell. Vesicles* **10**, e12142 (2021).
4. D. Gupta, O. P. B. Wiklander, A. Görgens, M. Conceição, G. Corso, X. Liang, Y. Seow, S. Balusu, U. Feldin, B. Bostancioglu, R. Jawad, D. R. Mamand, Y. X. F. Lee, J. Hean, I. Mäger, T. C. Roberts, M. Gustafsson, D. K. Mohammad, H. Sork, A. Backlund, P. Lundin, A. de Fougerolles, C. I. E. Smith, M. J. A. Wood, R. E. Vandenbroucke, J. Z. Nordin, S. El-Andaloussi, Amelioration of systemic inflammation via the display of two different decoy protein receptors on extracellular vesicles. *Nat. Biomed. Eng.* **5**, 1084–1098 (2021).
5. S. N. Hurwitz, M. A. Rider, J. L. Bundy, X. Liu, R. K. Singh, D. G. Meckes Jr., Proteomic profiling of NCI-60 extracellular vesicles uncovers common protein cargo and cancer type-specific biomarkers. *Oncotarget* **7**, 86999–87015 (2016).
6. H. Sork, G. Corso, K. Krjutskov, H. J. Johansson, J. Z. Nordin, O. P. B. Wiklander, Y. X. F. Lee, J. O. Westholm, J. Lehtiö, M. J. A. Wood, I. Mäger, S. El Andaloussi, Heterogeneity and interplay of the extracellular vesicle small RNA transcriptome and proteome. *Sci. Rep.* **8**, 10813 (2018).
7. K. O'Brien, K. Breyne, S. Ughetto, L. C. Laurent, X. O. Breakefield, RNA delivery by extracellular vesicles in mammalian cells and its applications. *Nat. Rev. Mol. Cell Biol.* **21**, 585–606 (2020).
8. F. Teng, M. Fussenegger, Shedding light on extracellular vesicle biogenesis and bioengineering. *Adv. Sci. (Weinh)* **8**, 2003505 (2021).
9. M. Chivet, C. Javalet, K. Laulagnier, B. Blot, F. J. Hemming, R. Sadoul, Exosomes secreted by cortical neurons upon glutamatergic synapse activation specifically interact with neurons. *J. Extracell. Vesicles* **3**, 24722 (2014).
10. M. Sancho-Albero, N. Navascués, G. Mendoza, V. Sebastián, M. Arruebo, P. Martín-Duque, J. Santamaría, Exosome origin determines cell targeting and the transfer of therapeutic nanoparticles towards target cells. *J. Nanobiotechnology* **17**, 16 (2019).
11. R. N. Hamzah, K. M. Alghazali, A. S. Biris, R. J. Griffin, Exosome traceability and cell source dependence on composition and cell-cell cross talk. *Int. J. Mol. Sci.* **22**, 5346 (2021).
12. O. P. B. Wiklander, J. Z. Nordin, A. O'Loughlin, Y. Gustafsson, G. Corso, I. Mäger, P. Vader, Y. Lee, H. Sork, Y. Seow, N. Heldring, L. Alvarez-Erviti, C. I. E. Smith, K. Le Blanc, P. Macchiarini, P. Jungebluth, M. J. A. Wood, S. E. Andaloussi, Extracellular vesicle in vivo biodistribution is determined by cell source, route of administration and targeting. *J. Extracell. Vesicles* **4**, 26316 (2015).
13. R. Poupardin, M. Wolf, D. Strunk, Adherence to minimal experimental requirements for defining extracellular vesicles and their functions. *Adv. Drug Deliv. Rev.* **176**, 113872 (2021).
14. D. Gupta, A. M. Zickler, S. El Andaloussi, Dosing extracellular vesicles. *Adv. Drug Deliv. Rev.* **178**, 113961 (2021).
15. C. Venugopal, C. Shamir, S. Senthilkumar, J. V. Babu, P. K. Sonu, K. J. Nishtha, K. S. Rai, S. K, A. Dhanushkodi, Dosage and passage dependent neuroprotective effects of exosomes derived from rat bone marrow mesenchymal stem cells: An in vitro analysis. *Curr. Gene Ther.* **17**, 379–390 (2017).
16. M. Albanese, Y.-F. A. Chen, C. Hüls, K. Gärtner, T. Tagawa, E. Mejias-Perez, O. T. Keppler, C. Göbel, R. Zeidler, M. Shein, A. K. Schütz, W. Hammerschmidt, MicroRNAs are minor constituents of extracellular vesicles that are rarely delivered to target cells. *PLOS Genet.* **17**, 1009951 (2021).
17. T. Driedonks, L. Jiang, B. Carlson, Z. Han, G. Liu, S. E. Queen, E. N. Shirk, O. Gololobova, Z. Liao, L. H. Nyberg, G. Lima, L. Paniushkina, M. Garcia-Contreras, K. Schonvisky, N. Castell, M. Stover, S. Guerrero-Martin, R. Richardson, B. Smith, V. Machairaki, C. P. Lai, J. M. Izzi, E. K. Hutchinson, K. A. M. Pate, K. W. Witwer, Pharmacokinetics and biodistribution of

extracellular vesicles administered intravenously and intranasally to *Macaca nemestrina*. *J. Extracell. Biol.* **1**, e59 (2022).
18. S. Bian, L. Zhang, L. Duan, X. Wang, Y. Min, H. Yu, Extracellular vesicles derived from human bone marrow mesenchymal stem cells promote angiogenesis in a rat myocardial infarction model. *J. Mol. Med.* **92**, 387–397 (2014).
19. G. J. Moon, J. H. Sung, D. H. Kim, E. H. Kim, Y. H. Cho, J. P. Son, J. M. Cha, O. Y. Bang, Application of mesenchymal stem cell-derived extracellular vesicles for stroke: Biodistribution and microRNA study. *Transl. Stroke Res.* **10**, 509–521 (2019).
20. L. Otero-Ortega, F. Laso-García, M. C. G.-d. Frutos, L. Diekhorst, A. Martínez-Arroyo, E. Alonso-López, M. L. García-Bermejo, M. Rodríguez-Serrano, M. Arrúe-Gonzalo, E. Díez-Tejedor, B. Fuentes, M. Gutiérrez-Fernández, Low dose of extracellular vesicles identified that promote recovery after ischemic stroke. *Stem Cell Res. Ther.* **11**, 70 (2020).
21. E. D. Sverdlov, Amedeo Avogadro's cry: What is 1 μg of exosomes? *Bioessays* **34**, 873–875 (2012).
22. A. Görgens, G. Corso, D. W. Hagey, R. Jawad Wiklander, M. O. Gustafsson, U. Felldin, Y. Lee, R. B. Bostancioglu, H. Sork, X. Liang, W. Zheng, D. K. Mohammad, S. I. van de Wakker, P. Vader, A. M. Zickler, D. R. Mamand, L. Ma, M. N. Holme, M. M. Stevens, O. P. B. Wiklander, S. El Andaloussi, Identification of storage conditions stabilizing extracellular vesicles preparations. *J. Extracell. Vesicles* **11**, e12238 (2022).
23. S. Picelli, O. R. Faridani, A. K. Björklund, G. Winberg, S. Sagasser, R. Sandberg, Full-length RNA-seq from single cells using Smart-seq2. *Nat. Protoc.* **9**, 171–181 (2014).
24. C. Loinard, A. Ribault, B. Lhomme, M. Benderitter, S. Flamant, S. Paul, V. Dubois, R. C. Lai, S. K. Lim, R. Tamarat, HuMSC-EV induce monocyte/macrophage mobilization to orchestrate neovascularization in wound healing process following radiation injury. *Cell Death Discov.* **9**, 38 (2023).
25. D. W. Hagey, E. Kvedaraite, M. Akber, A. Görgens, J. Javadi, T. Von Bahr Greenwood, C. Björklund, S. O. Åkefeldt, T. Hannegård-Hamrin, H. Arnell, K. Dobra, N. Herold, M. Svensson, S. El Andaloussi, J.-I. Henter, M. Lourda, Myeloid cells from Langerhans cell histiocytosis patients exhibit increased vesicle trafficking and an altered secretome capable of activating NK cells. *Haematologica* 10.3324/haematol.2022.282638 (2023).
26. W. Heusermann, J. Hean, D. Trojer, E. Steib, S. von Bueren, A. Graff-Meyer, C. Genoud, K. Martin, N. Pizzato, J. Voshol, D. V. Morrissey, S. E. Andaloussi, M. J. Wood, N. C. Meisner-Kober, Exosomes surf on filopodia to enter cells at endocytic hot spots, traffic within endosomes, and are targeted to the ER. *J. Cell Biol.* **213**, 173–184 (2016).
27. O. P. B. Wiklander, R. B. Bostancioglu, J. A. Welsh, A. M. Zickler, F. Murke, G. Corso, U. Felldin, D. W. Hagey, B. Evertsson, X. M. Liang, M. O. Gustafsson, D. K. Mohammad, C. Wiek, H. Hanenberg, M. Bremer, D. Gupta, M. Björnstedt, B. Giebel, J. Z. Nordin, J. C. Jones, S. El Andaloussi, A. Görgens, Systematic methodological evaluation of a multiplex bead-based flow cytometry assay for detection of extracellular vesicle surface signatures. *Front. Immunol.* **9**, 1326 (2018).
28. D. W. Hagey, D. Topcic, N. Kee, F. Reynaud, M. Bergsland, T. Perlmann, J. Muhr, CYCLIN-B1/2 and -D1 act in opposition to coordinate cortical progenitor self-renewal and lineage commitment. *Nat. Commun.* **11**, 2898 (2020).
29. D. W. Hagey, C. Zaouter, G. Combeau, M. A. Lendahl, O. Andersson, M. Huss, J. Muhr, Distinct transcription factor complexes act on a permissive chromatin landscape to establish regionalized gene expression in CNS stem cells. *Genome Res.* **26**, 908–917 (2016).
30. M. I. Love, W. Huber, S. Anders, Moderated estimation of fold change and dispersion for RNA-seq data with DESeq2. *Genome Biol.* **15**, 550 (2014).
31. H. Mi, A. Muruganujan, D. Ebert, X. Huang, P. D. Thomas, PANTHER version 14: More genomes, a new PANTHER GO-slim and improvements in enrichment analysis tools. *Nucleic Acids Res.* **27**, D419–D426 (2019).
32. J. P. Bost, O. Saher, D. Hagey, D. R. Mamand, X. Liang, W. Zheng, G. Corso, O. Gustafsson, A. Görgens, C. E. Smith, R. Zain, S. El Andaloussi, D. Gupta, Growth media conditions influence the secretion route and release levels of engineered extracellular vesicles. *Adv. Healthc. Mater.* **11**, e2101658 (2022).
33. J. P. Bost, M. Ojansivu, M. J. Munson, E. Wesén, A. Gallud, D. Gupta, O. Gustafsson, O. Saher, J. Rädler, S. G. Higgins, T. Lehto, M. N. Holme, A. Dahlén, O. Engkvist, P. E. Strömstedt, S. Andersson, C. I. Edvard Smith, M. M. Stevens, E. K. Esbjörner, A. Collén, S. El Andaloussi, Novel endosomolytic compounds enable highly potent delivery of antisense oligonucleotides. *Commun. Biol.* **5**, 185 (2022).
34. L. von Chamier, R. F. Laine, J. Jukkala, C. Spahn, D. Krentzel, E. Nehme, M. Lerche, S. Hernández-Pérez, P. K. Mattila, E. Karinou, S. Holden, A. C. Solak, A. Krull, T.-O. Buchholz, M. L. Jones, L. A. Royer, C. Leterrier, Y. Shechtman, F. Jug, M. Heilemann, G. Jacquemet, R. Henriques, Democratising deep learning for microscopy with ZeroCostDL4Mic. *Nat. Commun.* **12**, 2276 (2021).
35. Tabula Muris Consortium; Overall Coordination; Logistical Coordination; Organ Collection and Processing; Library Preparation and Sequencing; Computational Data Analysis; Cell Type Annotation; Writing Group; Supplemental Text Writing Group; Principal Investigators, Single-cell transcriptomics of 20 mouse organs creates a *Tabula Muris*. *Nature* **562**, 367–372 (2018).
36. T. L. Deem, J. B. Collins, M. H. DeVost, C. O. Parker, S. C. Saroka, R. J. Zoldork, F. Gutierrez, J. M. Russell, C. S. Lantz, Assessment of faithful interleukin-3 production by novel bicistronic interleukin-3 reporter mice. *Immunol. Lett.* **221**, 18–26 (2020).
37. A. R. Silini, R. Di Pietro, I. Lang-Olip, F. Alviano, A. Banerjee, M. Basile, V. Borutinskaite, G. Eissner, A. Gellhaus, B. Giebel, Y.-C. Huang, A. Janev, M. E. Kreft, N. Kupper, A. C. Abadía-Molina, E. G. Olivares, A. Pandolfi, A. Papait, M. Pozzobon, C. Ruiz-Ruiz, O. Soritau, S. Susman, D. Szukiewicz, A. Weidinger, S. Wolbank, B. Huppertz, O. Parolini, Perinatal derivatives: Where do we stand? A roadmap of the human placenta and consensus for tissue and cell nomenclature. *Front. Bioeng. Biotechnol.* **8**, 610544 (2020).
38. J. Z. Nordin, R. B. Bostancioglu, G. Corso, S. El Andaloussi, Tangential flow filtration with or without subsequent bind-elute size exclusion chromatography for purification of extracellular vesicles. *Methods Mol. Biol.* **1953**, 287–299 (2019).
39. R. A. Dragovic, C. Gardiner, A. S. Brooks, D. S. Tannetta, D. J. P. Ferguson, P. Hole, B. Carr, C. W. G. Redman, A. L. Harris, P. J. Dobson, P. Harrison, I. L. Sargent, Sizing and phenotyping of cellular vesicles using nanoparticle tracking analysis. *Nanomedicine* **7**, 780–788 (2011).
40. M. Ovesný, P. Křížek, J. Borkovec, Z. Švindrych, G. M. Hagen, ThunderSTORM: A comprehensive ImageJ plug-in for PALM and STORM data analysis and super-resolution imaging. *Bioinformatics* **30**, 2389–2390 (2014).

Acknowledgments: We acknowledge X. Li, J. Liung, and S. Gustavsson of the Single Cell Core Facility Karolinska Flemingsberg, as well as A. Zickler for editorial commentary. Schematics were produced using BioRender. **Funding:** A.G. is an International Society for Advancement of Cytometry Marylou Ingram Scholar. O.S. was supported by a PhD grant from the Egyptian Ministry of Higher Education. M.O. is supported by the Swedish Foundation of Strategic Research (FormulaEx IRC15-0065). S.E.A. is supported by H2020 EXPERT (no. 825828), ERC CoG DELIVER (no. 101001374), the Swedish Medical Research Council (VR-Med 2020-01322), and EVOX Therapeutics Ltd. D.W.H. is supported by the Swedish Cancer Foundation (#21 0378 PT), the Karolinska Institute Research Foundation, and Magnus Bergvalls Foundation. **Author contributions:** Conceptualization: D.W.H., A.G., and S.E.A. Methodology: D.W.H., M.O., B.R.B., O.S., J.P.B., R.G., M.S., and A.G. Investigation: D.W.H., M.O., B.R.B., O.S., J.P.B., M.O.G., and A.G. Visualization: D.W.H., M.O., B.R., and A.G. Supervision: D.W.H., A.G., M.M.S., and S.E.A. Writing–original draft: D.W.H., M.O., B.R.B., R.G., D.G., A.G., and S.E.A. Writing–review and editing: D.W.H., A.G., and S.E.A. **Competing interests:** M.M.S. is invested in, on boards of, consults for, and conducts research funded by companies related to the biomaterials field, as well as being co-inventor on multiple patents and patent applications describing nanomaterials for therapeutic delivery, regenerative medicine, and diagnostics. A.G. and S.E.A. consult for and have financial stakes in Evox Therapeutics, The other authors declare that they have no competing interests. **Data and materials availability:** All data needed to evaluate the conclusions in the paper are present in the paper and/or the Supplementary Materials. Raw sequencing data used in this work has been uploaded to the NCBI SRA database under the accession number PRJNA910343. Graph data and gene count tables, as well as template scripts, are available via Dryad doi: 10.5061/dryad.b2rbnzsmj.

Submitted 14 February 2023
Accepted 31 July 2023
Published 1 September 2023
10.1126/sciadv.adh1168
