# Supplementary material for: The cellular response to extracellular vesicles is dependent on their cell source and dose
Source: Sci Adv. 2023 Sep 1;9(35):eadh1168. doi: 10.1126/sciadv.adh1168 (PMC11629882; doi:10.1126/sciadv.adh1168)
Supplement: Supplementary file 2 — Figs. S1 to S13 Legends for tables S1 to S3 [file sciadv.adh1168_sm.pdf]

Science Advances
AAAS

Supplementary Materials for

The cellular response to extracellular vesicles is dependent on their cell source and dose

Daniel W. Hagey *et al.*

Corresponding author: Daniel W. Hagey, daniel.hagey@ki.se

Sci. Adv. **9**, eadh1168 (2023)
DOI: 10.1126/sciadv.adh1168

The PDF file includes:

Figs. S1 to S12
Legends for tables S1 to S3

Other Supplementary Material for this manuscript includes the following:

Tables S1 to S3

Supplementary Material

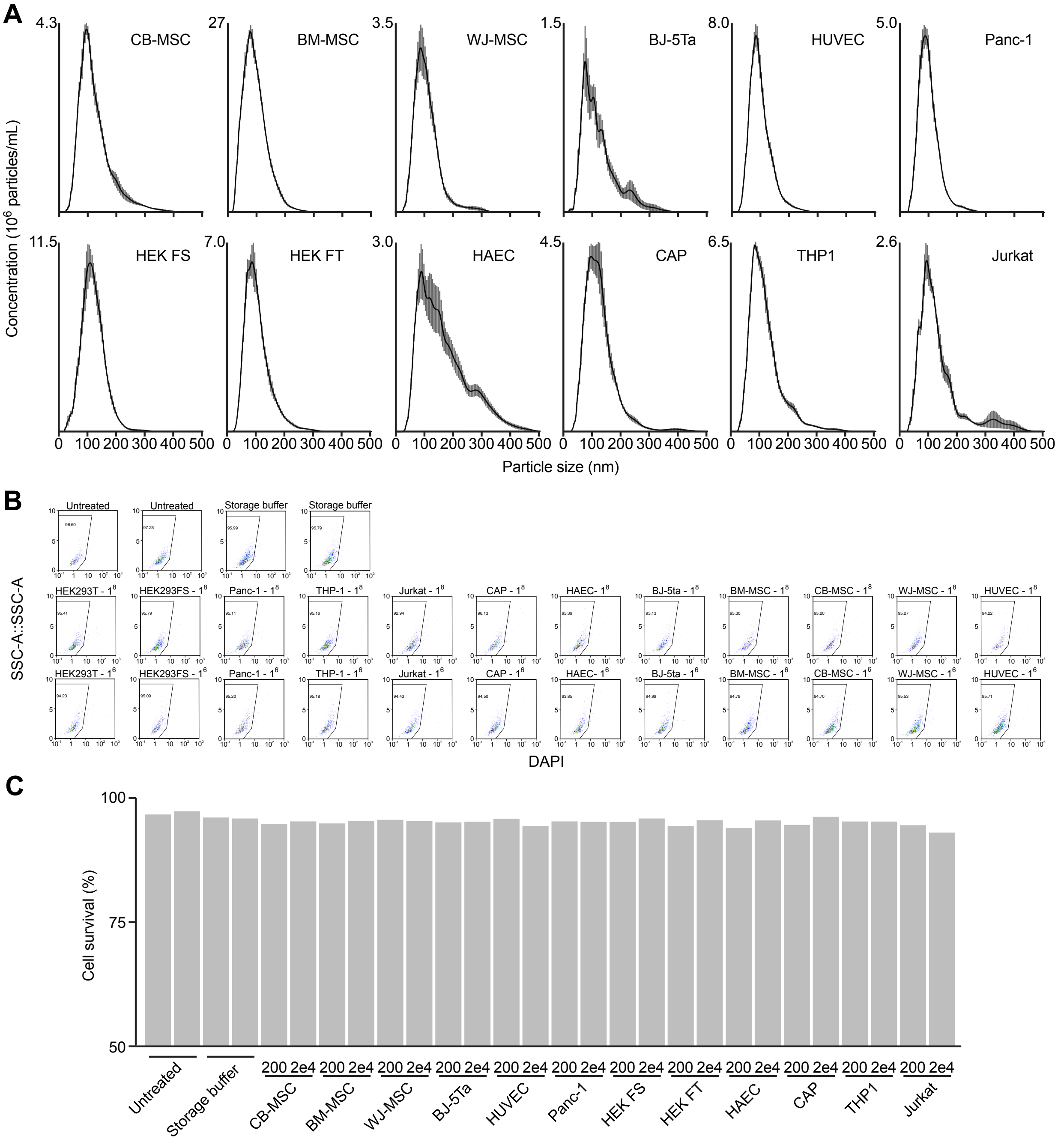

Supplementary Figure 1. EVs do not affect cell survival. (A) Nanoparticle tracking analysis size distribution graphs of EVs isolated from each cell line. (B) Comparison of cell viability of fibroblasts after treatment with storage buffer or 200 or 2e4 EVs per cell, as assayed by flow cytometry. (C) Quantification of flow cytometry cell viability data at two doses of EVs from each cell source versus controls (storage buffer and untreated cells).

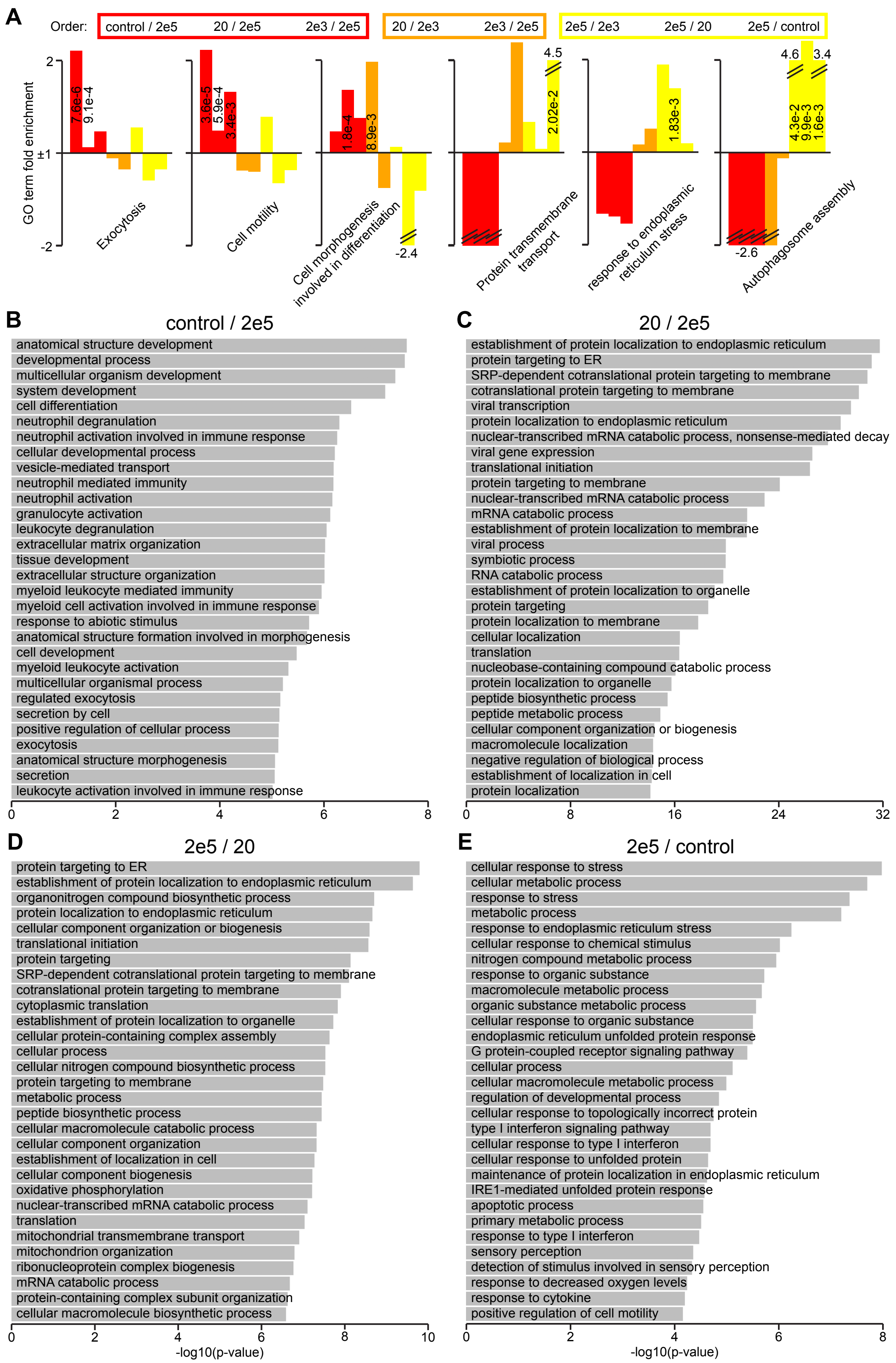
A
Order:
control / 2e5
20 / 2e5
2e3 / 2e5
20 / 2e3
2e3 / 2e5
2e5 / 2e3
2e5 / 20
2e5 / control
GO term fold enrichment
2
±1
-2
7.6e-6
9.1e-4
Exocytosis
3.6e-5
5.9e-4
3.4e-3
Cell motility
1.8e-4
8.9e-3
-2.4
Cell morphogenesis involved in differentiation
4.5
2.02e-2
Protein transmembrane transport
1.83e-3
response to endoplasmic reticulum stress
4.6
3.4
4.3e-2
9.9e-3
1.6e-3
-2.6
Autophagosome assembly
B
control / 2e5
anatomical structure development
developmental process
multicellular organism development
system development
cell differentiation
neutrophil degranulation
neutrophil activation involved in immune response
cellular developmental process
vesicle-mediated transport
neutrophil mediated immunity
neutrophil activation
granulocyte activation
leukocyte degranulation
extracellular matrix organization
tissue development
extracellular structure organization
myeloid leukocyte mediated immunity
myeloid cell activation involved in immune response
response to abiotic stimulus
anatomical structure formation involved in morphogenesis
cell development
myeloid leukocyte activation
multicellular organismal process
regulated exocytosis
secretion by cell
positive regulation of cellular process
exocytosis
anatomical structure morphogenesis
secretion
leukocyte activation involved in immune response
0
2
4
6
8
C
20 / 2e5
establishment of protein localization to endoplasmic reticulum
protein targeting to ER
SRP-dependent cotranslational protein targeting to membrane
cotranslational protein targeting to membrane
viral transcription
protein localization to endoplasmic reticulum
nuclear-transcribed mRNA catabolic process, nonsense-mediated decay
viral gene expression
translational initiation
protein targeting to membrane
nuclear-transcribed mRNA catabolic process
mRNA catabolic process
establishment of protein localization to membrane
viral process
symbiotic process
RNA catabolic process
establishment of protein localization to organelle
protein targeting
protein localization to membrane
cellular localization
translation
nucleobase-containing compound catabolic process
protein localization to organelle
peptide biosynthetic process
peptide metabolic process
cellular component organization or biogenesis
macromolecule localization
negative regulation of biological process
establishment of localization in cell
protein localization
0
8
16
24
32
D
2e5 / 20
protein targeting to ER
establishment of protein localization to endoplasmic reticulum
organonitrogen compound biosynthetic process
protein localization to endoplasmic reticulum
cellular component organization or biogenesis
translational initiation
protein targeting
SRP-dependent cotranslational protein targeting to membrane
cotranslational protein targeting to membrane
cytoplasmic translation
establishment of protein localization to organelle
cellular protein-containing complex assembly
cellular process
cellular nitrogen compound biosynthetic process
protein targeting to membrane
metabolic process
peptide biosynthetic process
cellular macromolecule catabolic process
cellular component organization
establishment of localization in cell
cellular component biogenesis
oxidative phosphorylation
nuclear-transcribed mRNA catabolic process
translation
mitochondrial transmembrane transport
mitochondrion organization
ribonucleoprotein complex biogenesis
mRNA catabolic process
protein-containing complex subunit organization
cellular macromolecule biosynthetic process
0
2
4
6
8
10
-log10(p-value)
E
2e5 / control
cellular response to stress
cellular metabolic process
response to stress
metabolic process
response to endoplasmic reticulum stress
cellular response to chemical stimulus
nitrogen compound metabolic process
response to organic substance
macromolecule metabolic process
organic substance metabolic process
cellular response to organic substance
endoplasmic reticulum unfolded protein response
G protein-coupled receptor signaling pathway
cellular process
cellular macromolecule metabolic process
regulation of developmental process
cellular response to topologically incorrect protein
type I interferon signaling pathway
cellular response to type I interferon
cellular response to unfolded protein
maintenance of protein localization in endoplasmic reticulum
IRE1-mediated unfolded protein response
apoptotic process
primary metabolic process
response to type I interferon
sensory perception
detection of stimulus involved in sensory perception
response to decreased oxygen levels
response to cytokine
positive regulation of cell motility
0
2
4
6
8
-log10(p-value)

Supplementary Figure 2. Gene ontology analysis of genes differentially expressed in all samples treated at different doses of EVs. A) Gene ontology term fold enrichment for genes upregulated in control over cells treated with 2e5 EVs, 20 over 2e5 EV per cell treated samples, 2e3 over 2e5 EV per cell treated samples (all red), 20 over 2e3 EV per cell treated samples, 2e3 over 20 EV per cell treated samples (both orange), 2e5 over 2e3 EV per cell treated samples, 2e5 over 20 EV per cell treated samples or 2e5 EV per cell treated over control samples (all yellow). P-values for statistically significant terms are inset and negative values without fold change listed show no enrichment. B-E) P-values of the thirty most significant gene ontology terms for genes overrepresented in control over 2e5 EV per cell treated samples (B), 20 over 2e5 EV per cell treated samples (C), 2e5 over 20 EV per cell treated samples (D) or 2e5 EV per cell treated over control samples (E).

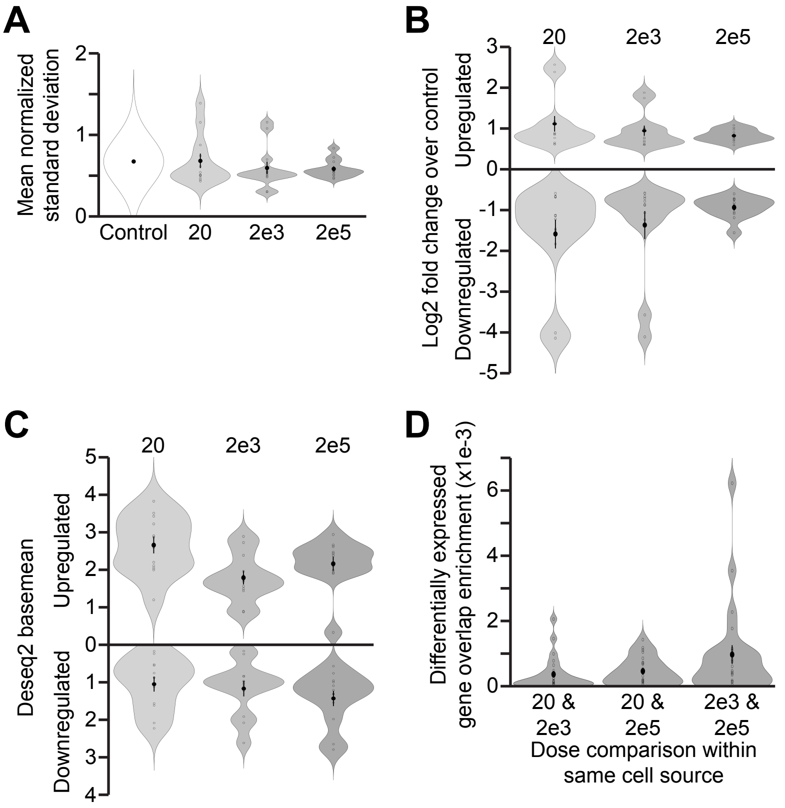

Supplementary Figure 3. Genes dysregulated by individual EV types have similar structural properties regardless of EV dose. A) Violin plots of mean normalized standard deviation of genes differentially expressed after treatment with different doses of EVs. B) Log2 fold change over control samples of genes differentially expressed after treatment with different doses of EVs. C) Expression levels of genes differentially expressed after treatment with different doses of EVs. D) Overlap enrichment scores between genes differentially expressed after treatment with different types of EVs at different doses. Sample mean is shown as a large solid dot, standard error as a horizontal line and individual data points as rings.

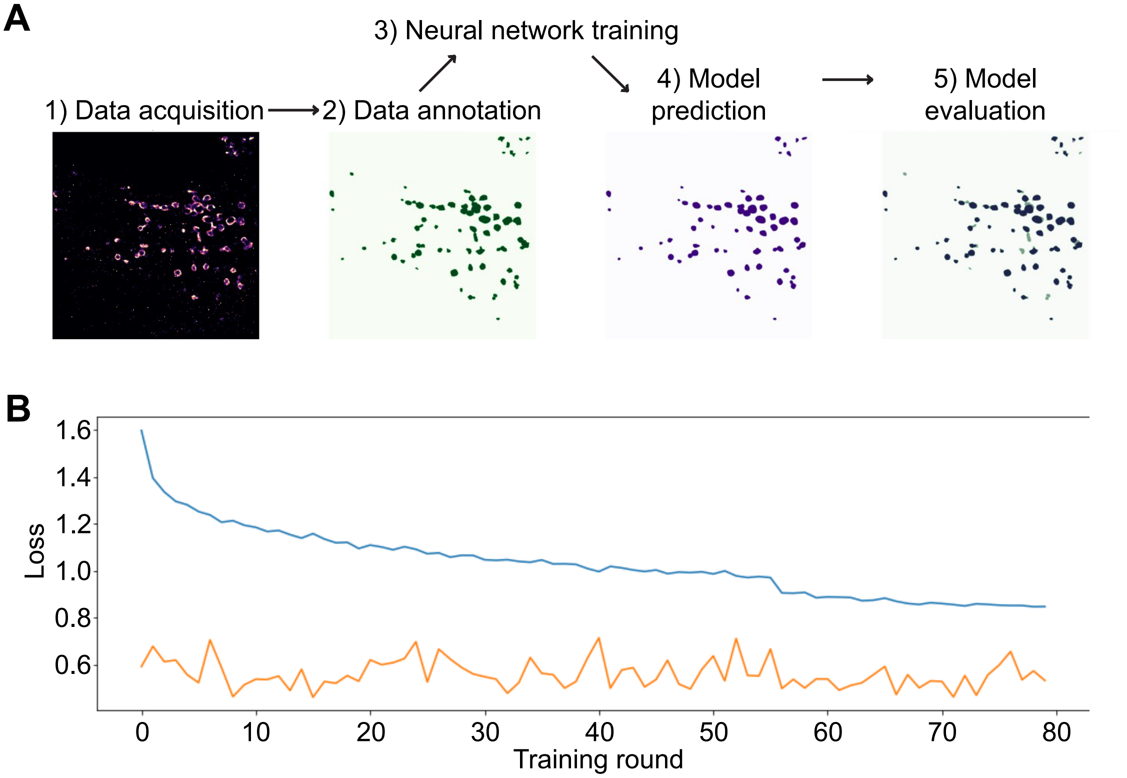

C

image #	Prediction v. GT IoU	false pos.	true pos.	false neg.	precision	recall	accuracy
1	0.799	1	62	14	0.984	0.816	0.805
2	0.835	8	121	12	0.938	0.91	0.858
3	0.84	6	52	6	0.897	0.897	0.812
4	0.687	1	80	31	0.988	0.721	0.714

Supplementary Figure 4. Overview of the deep learning approach and model quality control for the quantification of lysosome sizes. A) Schematic of the quantification workflow from the STORM data acquisition to the creation and evaluation of the deep learning model. B) Model evaluation during the training process by measurement of the error (loss) between the model predictions and manually annotated objects. The loss is presented separately for the training and validation data sets as a function of the training round (epoch). C) Evaluation of the model quality post-training with four validation images (1-4). These images were not used for the training/validation during the training. IoU was calculated for pixels, and precision, recall and accuracy for the identified objects. GT = ground truth (manually annotated objects). IoU = intersection over union (overlap of the manually annotated objects and the objects predicted by the model).

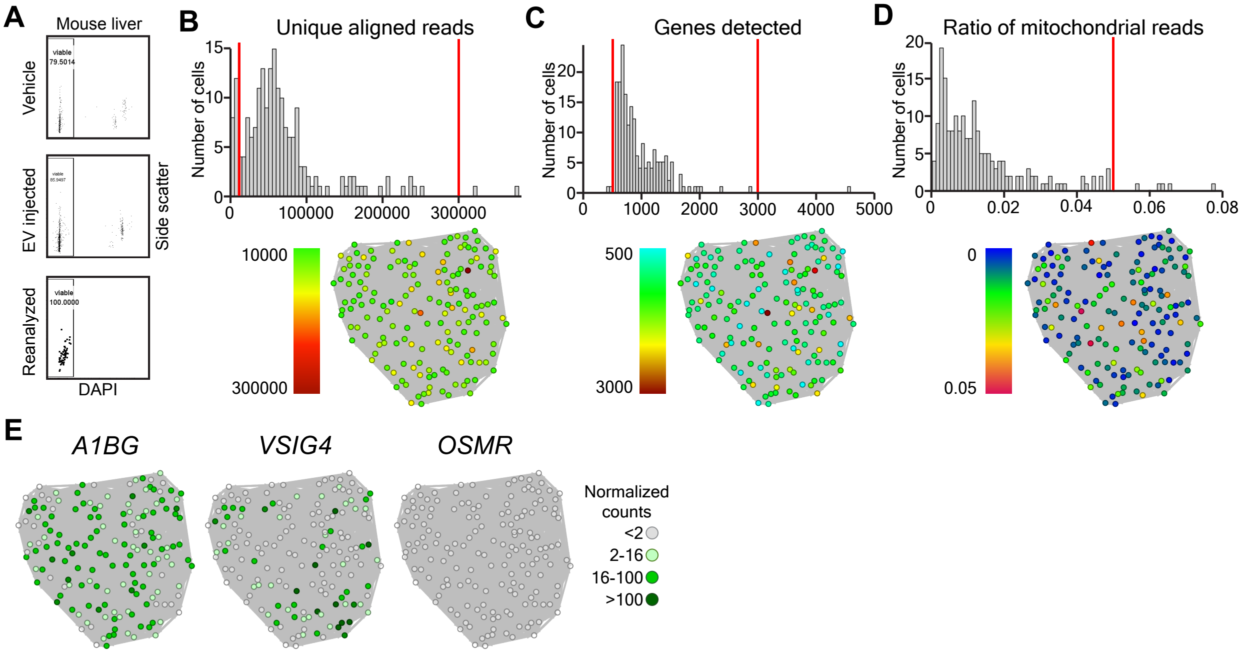

Supplementary Figure 5. Single cell RNA-sequencing QC strategy. A) FACS dot plots showing gating of DAPI-negative single cells from dissociated liver following injection with Vehicle, CD63-mNG EVs. mNeonGreen positive cells were reanalyzed for DAPI to ensure viability. B-D) Histograms showing all cells', and tSNE-NN maps showing cells passing QC, total mapped reads (B), detected genes (C) and ratio of mitochondrial reads (D) and the cutoffs (red lines) used to separate cells not passing QC. E) tSNE-NN maps coloured based on the expression of a hepatocyte (*A1BG*), Kuppfer (*VSIG4*) or endothelial cell (*OSMR*) marker.

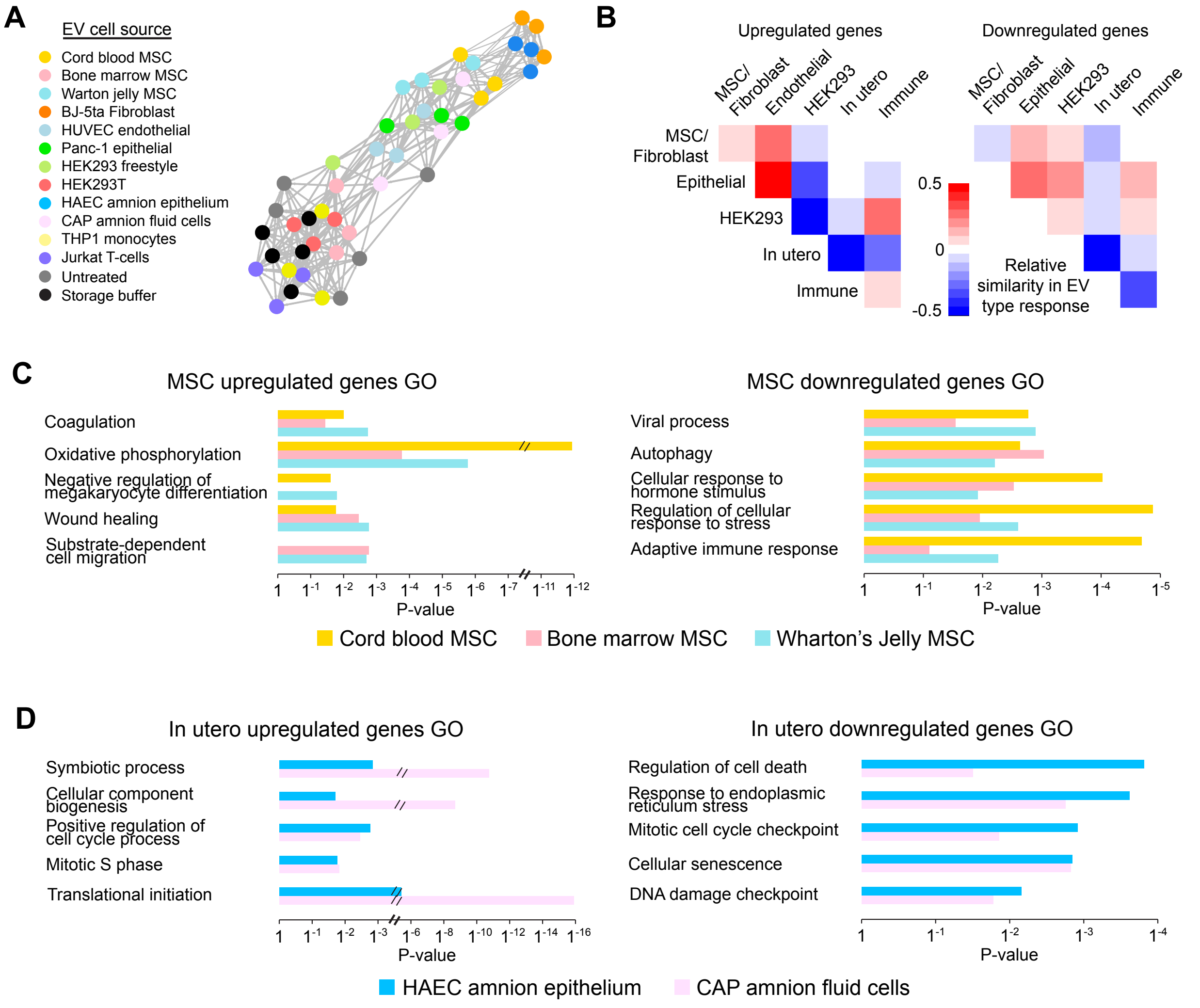

Supplementary Figure 6. Expanded analysis of transcriptional responses specific to EV cell source. A) tSNE-NN map from Figure 4A colored by the type of EVs they were treated with. B) Heatmaps of the relative similarity between up- or downregulated genes in fibroblasts based on the cell source group of the EVs they were treated with. C-D) P-values of selected gene ontology terms enriched in fibroblasts treated with MSC- (C) or in utero-derived (D) EVs.

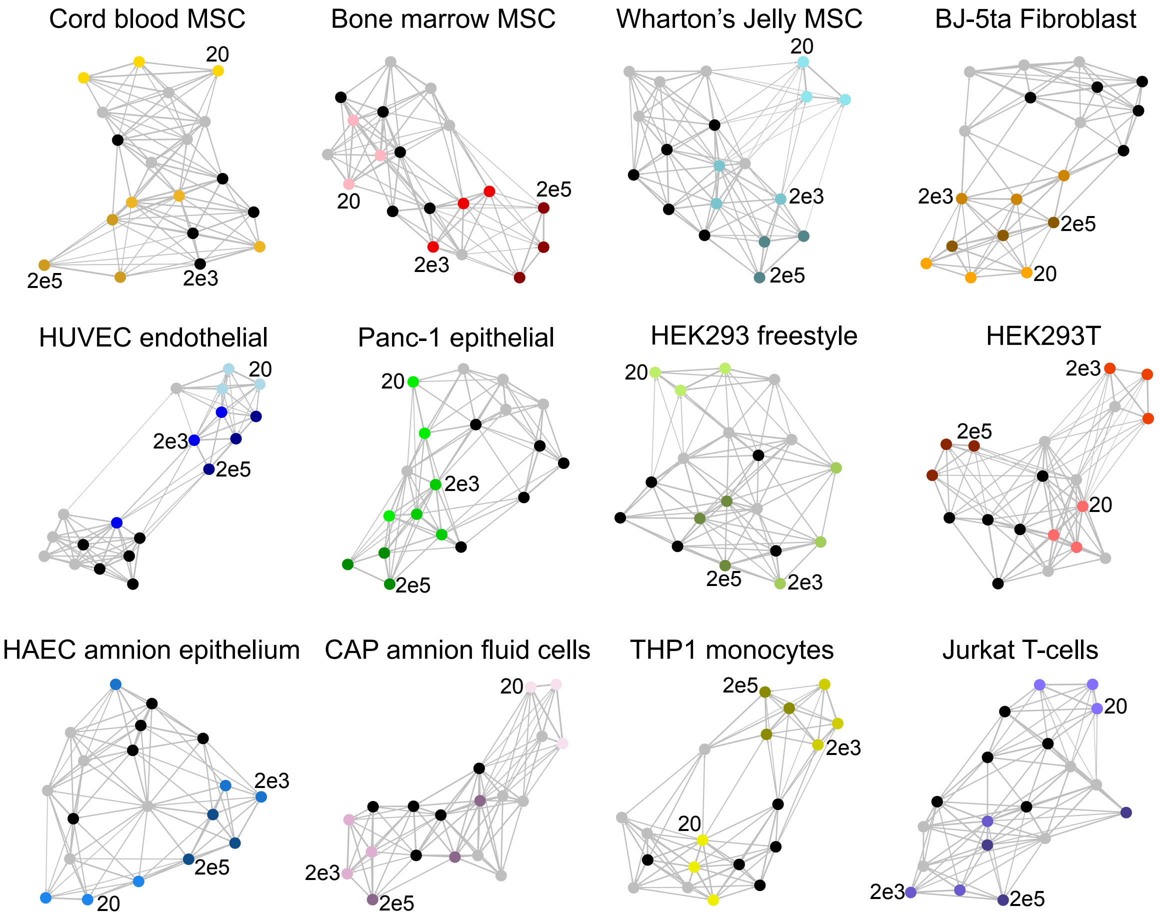

Supplementary Figure 7. Separate mapping of control and different EV type treated fibroblasts. Separate tSNE-NN maps of control fibroblast transcriptomes and those treated with each of the different types of EVs at doses of 20, 2e3 and 2e5 EVs per cell.

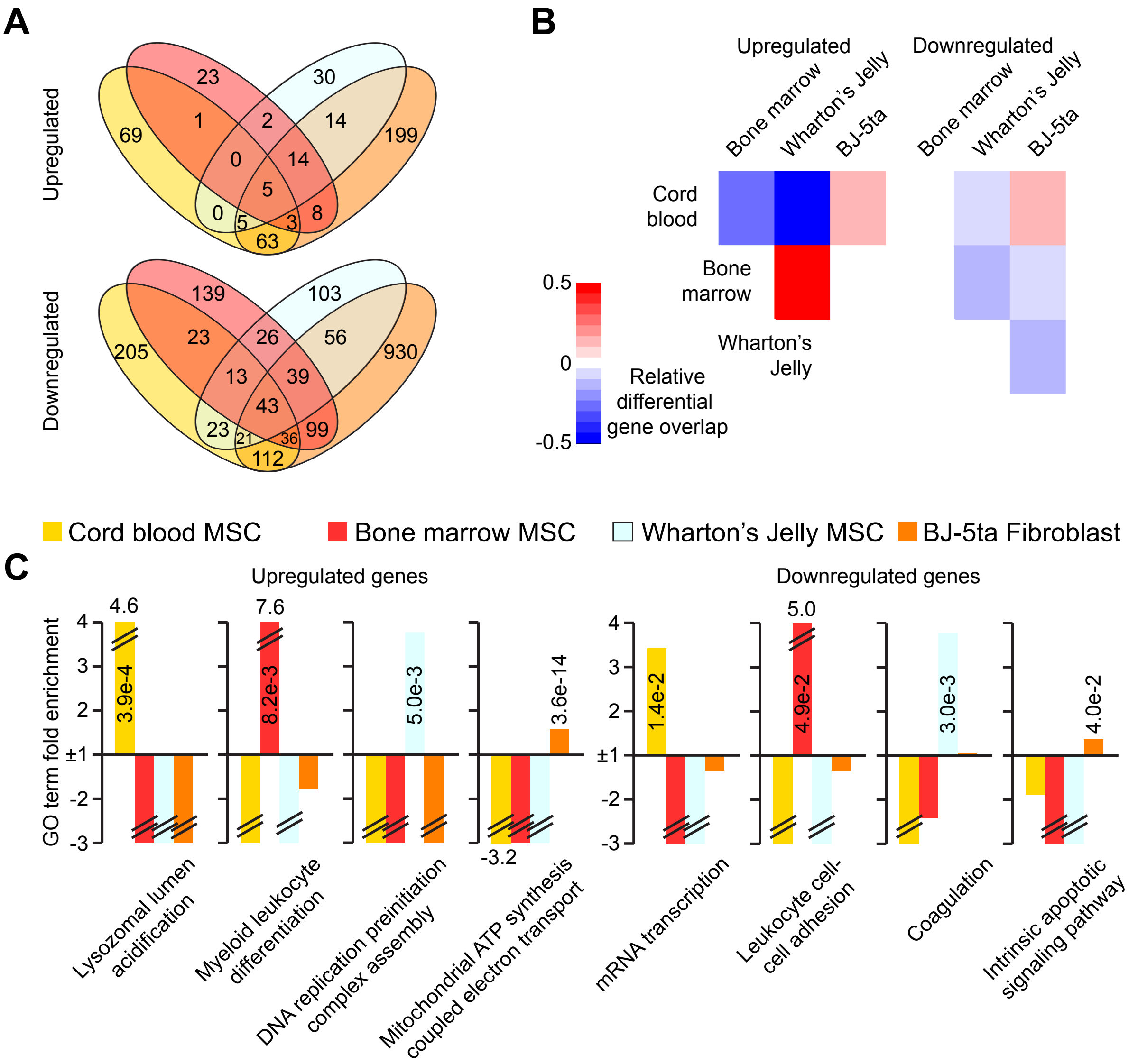

Supplementary Figure 8. Specific effects of the different types of MSC and fibroblast EVs. A) Venn diagrams of the genes robustly up- and downregulated by cord blood, bone marrow and Wharton's Jelly MSCs, as well as BJ-5ta fibroblasts from Figure 4. B) Heatmap of the relative overlap enrichment between the genes up- and downregulated by the different MSC and fibroblast EVs. C) Gene ontology term fold enrichment of genes uniquely up- or downregulated by a single type of MSC or fibroblast EV. P-values for statistically significant terms are inset and negative values without fold change listed show no enrichment.

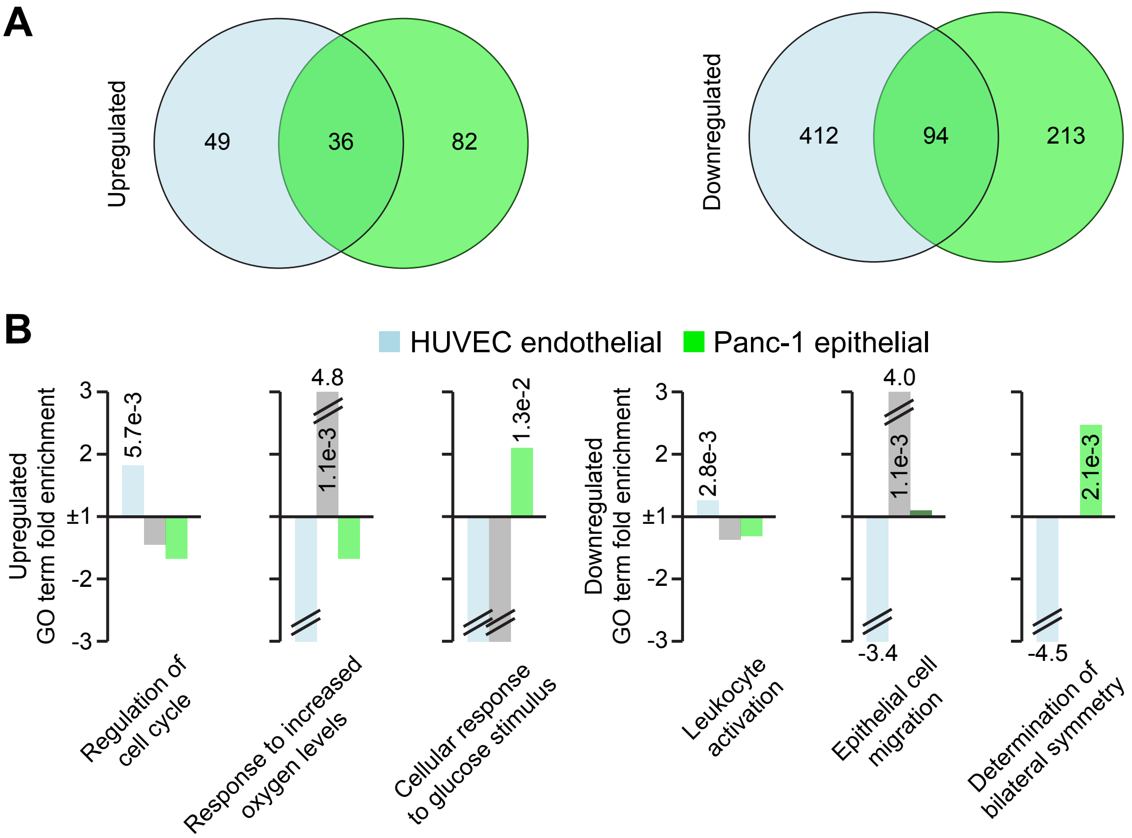

Supplementary Figure 9. Specific effects of the different types of epithelial cell EVs. A) Venn diagrams of the genes robustly up- and downregulated by HUVEC and Panc-1 epithelial cells from Figure 4. B) Gene ontology term fold enrichment of genes uniquely or commonly up- or downregulated by HUVEC and Panc-1 EVs. P-values for statistically significant terms are inset and negative values without fold change listed show no enrichment.

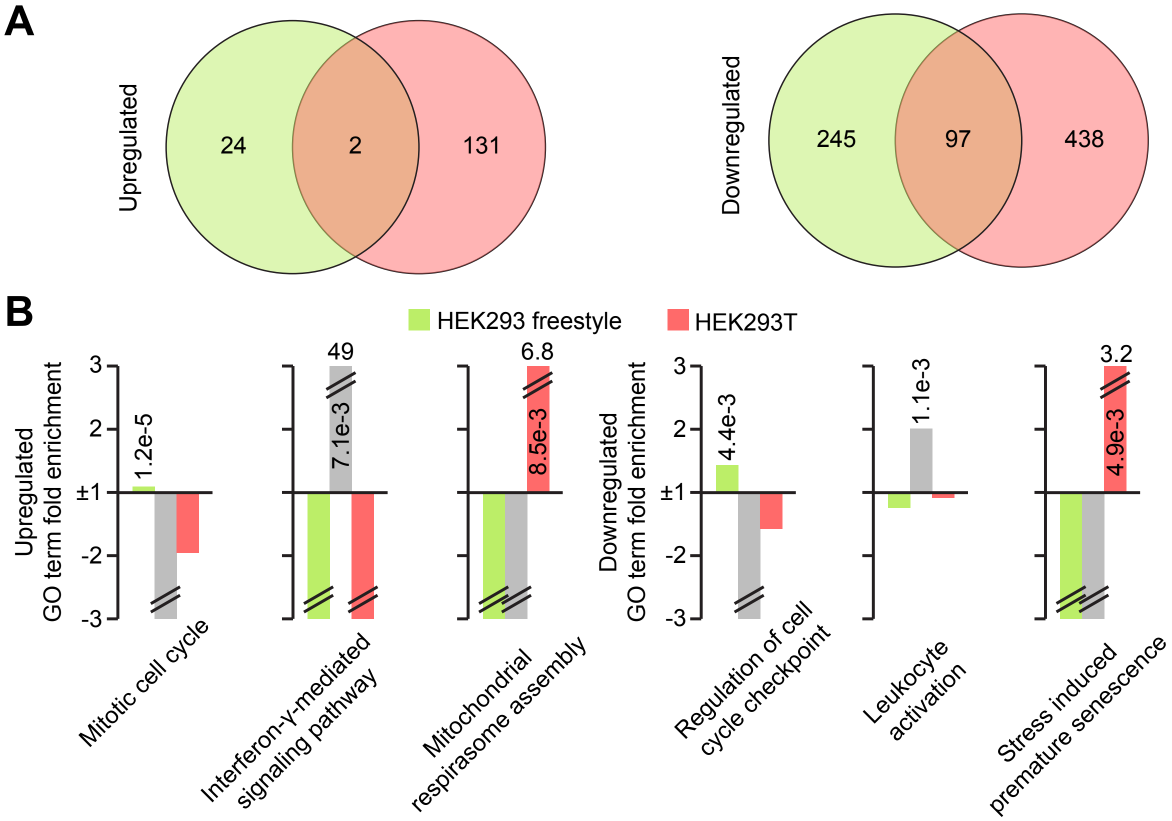

Supplementary Figure 10. Specific effects of the different types of HEK293 cell EVs. A) Venn diagrams of the genes robustly up- and downregulated by HEK293 freestyle and HEK293T cells from Figure 4. B) Gene ontology term fold enrichment of genes uniquely or commonly up- or downregulated by HEK293 freestyle and HEK293T EVs. P-values for statistically significant terms are inset and negative values without fold change listed show no enrichment.

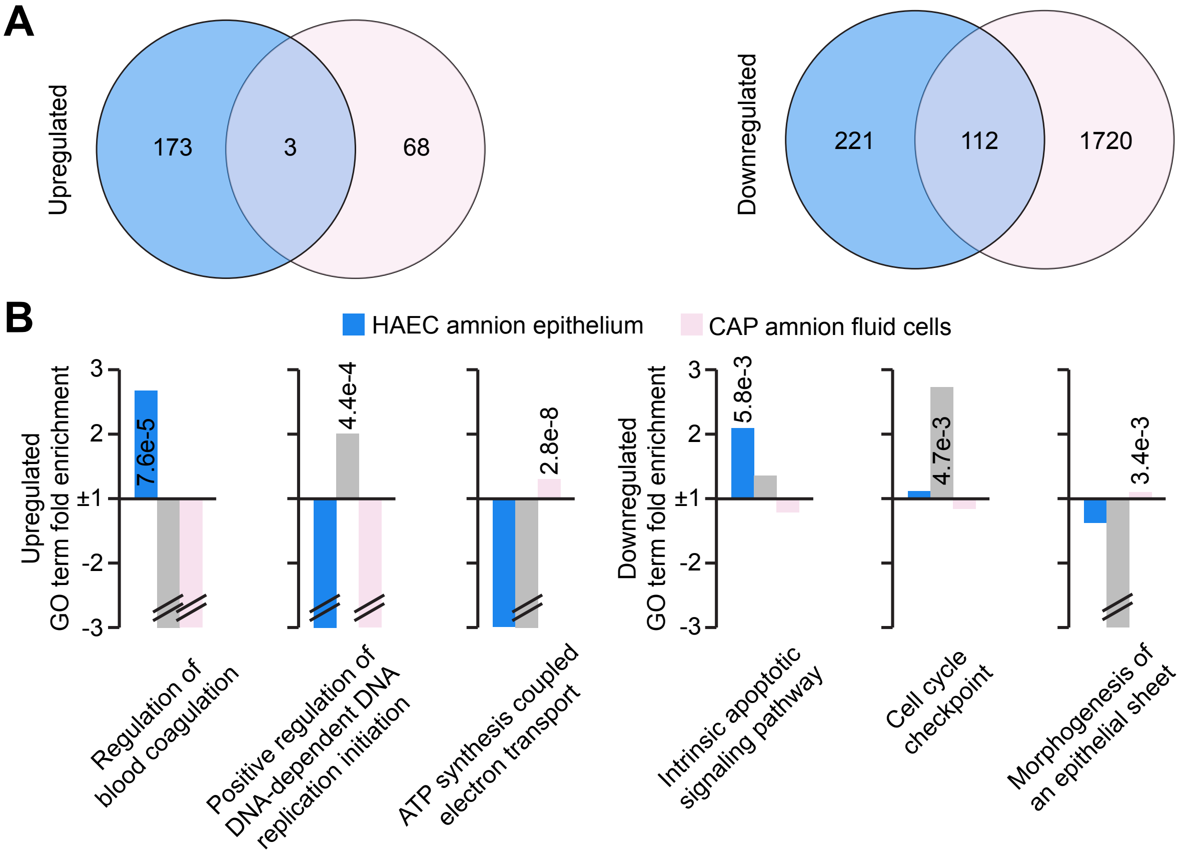

Supplementary Figure 11. Specific effects of the different types of in utero cell EVs. A) Venn diagrams of the genes robustly up- and downregulated by HAEC and CAP cells from Figure 4. B) Gene ontology term fold enrichment of genes uniquely or commonly up- or downregulated by HAEC and CAP EVs. P-values for statistically significant terms are inset and negative values without fold change listed show no enrichment.

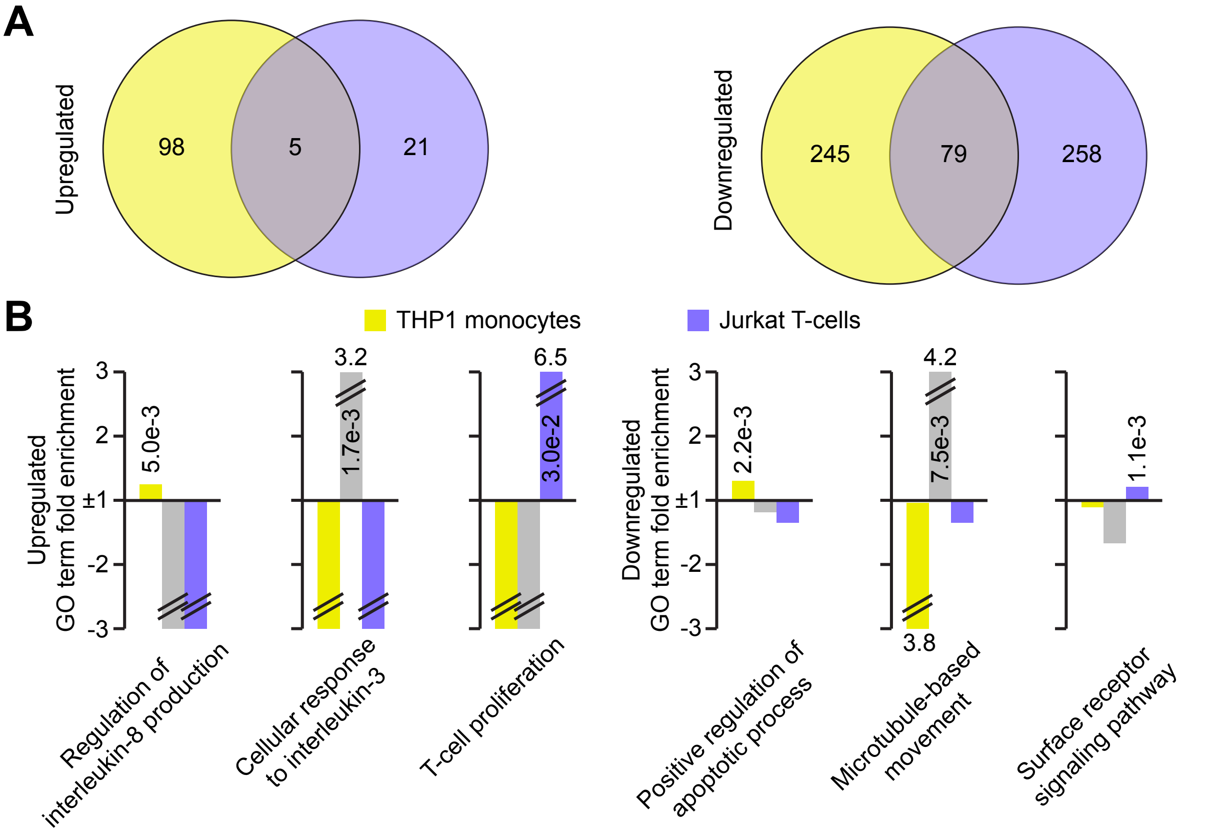

Supplementary Figure 12. Specific effects of the different types of immune cell EVs. A) Venn diagrams of the genes robustly up- and downregulated by THP1 and Jurkat cells from Figure 4. B) Gene ontology term fold enrichment of genes uniquely or commonly up- or downregulated by THP1 and Jurkat EVs. P-values for statistically significant terms are inset and negative values without fold change listed show no enrichment.

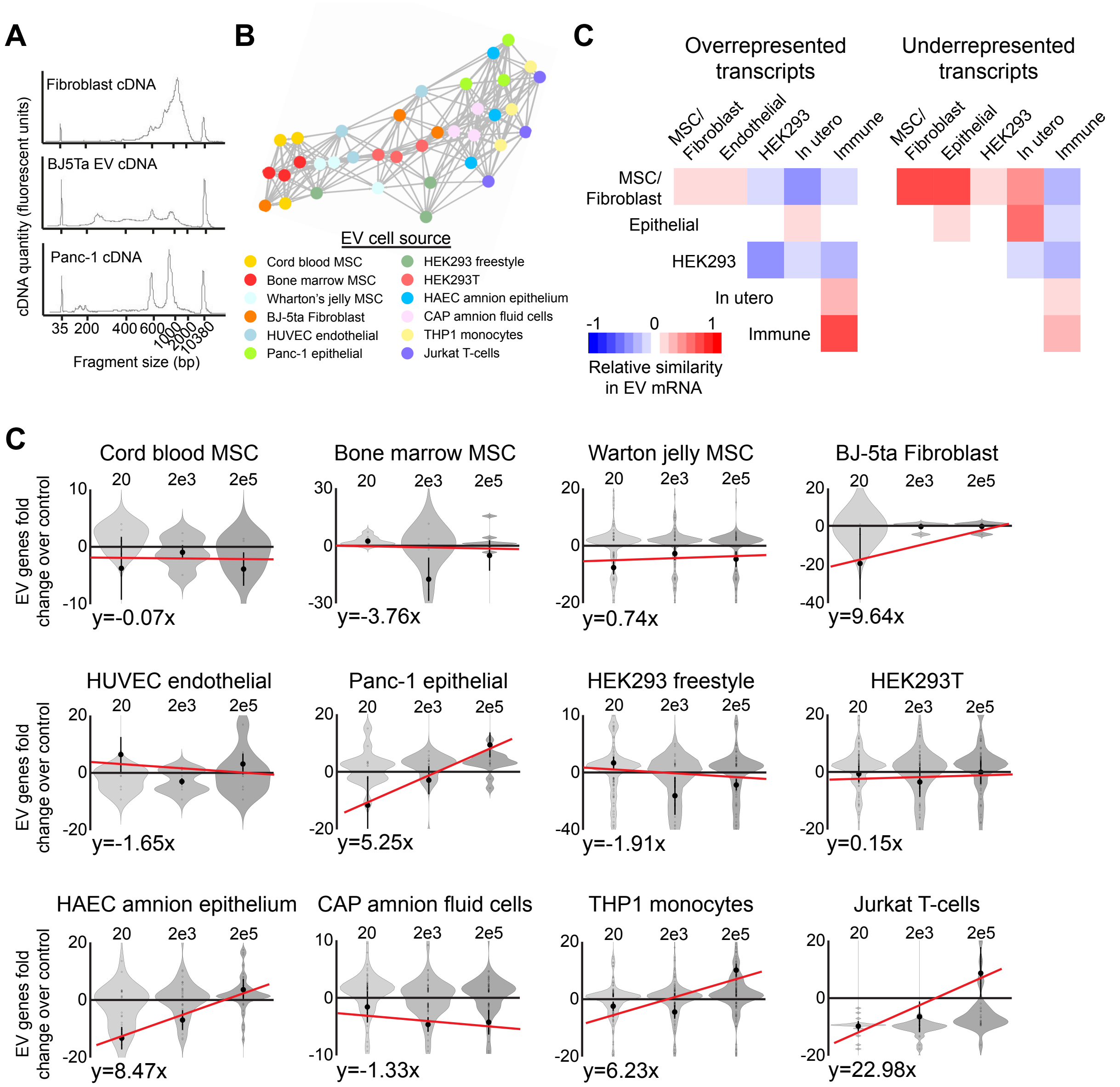

Supplementary Figure 13. Confirmation of EV transcript delivery. A) Bioanalyzed HS DNA traces showing cDNA obtained from primary human fibroblast cells, BJ5Ta fibroblast EVs and Panc-1 EVs. B) tSNE-NN map from Figure 5A colored by their cell source. C) Heatmaps of the relative similarity between over- or underrepresented transcripts in EVs from different cell source groups. D) Violin plots of the fold difference in fibroblast expression of transcripts specific to each type of EV over their basal levels in control fibroblasts, when treated with 20, 2e3 or 2e5 EVs per cell. Sample mean is shown as a large solid dot, standard error as a horizontal line and individual data points as rings. Best fit line is overlayed in red, with the slope of each line displayed below.

Table 1: Genes regulated by ≥5 cell source EVs when comparing control cells at one dose

Table 1. Genes regulated by ≥5 cell source EVs when comparing control cells at one

Down in 20 E	Up in 20 EV c	Down in 2e5 I	Up in 2e5 Dose
ENSG000001	ENSG000001	ENSG000001	ENSG00000135074
ENSG00000C	ENSG000001	ENSG000001	ENSG00000222041
ENSG000002	ENSG000001	ENSG000001	ENSG00000106366
ENSG00000C	ENSG000001	ENSG000001	ENSG00000117152
ENSG000001	ENSG000001	ENSG000001	ENSG00000117525
ENSG000001	ENSG000001	ENSG000001	ENSG00000138814
ENSG000001	ENSG000001	ENSG000001	ENSG00000172965
ENSG000002	ENSG000001	ENSG000001	ENSG00000115541
ENSG00000C	ENSG000002	ENSG000001	ENSG00000118181
ENSG000001	ENSG000002	ENSG000001	ENSG00000119335
ENSG000001	ENSG000002	ENSG000001	ENSG00000125148
ENSG000001	ENSG000002	ENSG000002	ENSG00000143977
ENSG000001	ENSG000002	ENSG000001	ENSG00000161960
ENSG00000163453		ENSG000001	ENSG00000197780
ENSG00000169902		ENSG000001	ENSG00000277957
ENSG00000175166		ENSG000001	ENSG00000280721
ENSG00000179632		ENSG000001	ENSG00000044574
ENSG00000198542		ENSG000001	ENSG00000086062
ENSG00000004142		ENSG000001	ENSG00000092820
ENSG00000022840		ENSG000001	ENSG00000099139
ENSG00000072778		ENSG000001	ENSG00000107796
ENSG00000079931		ENSG000001	ENSG00000108953
ENSG00000090060		ENSG000001	ENSG00000117519
ENSG00000090520		ENSG000001	ENSG00000128656
ENSG00000100075		ENSG000001	ENSG00000136603

Abridged

Table 2: Genes repeatedly upregulated by EVs from each cell source

Table 1. Genes upregulated in fibroblasts by EVs from different cell lines at two different doses.

Cord blood MSC	Bone marrow MSC	Whaton's jelly MSC	BJ-5ta	HUVEC	Panc-1
ENSG00000066136	ENSG00000166224	ENSG00000117152	ENSG00000143549	ENSG00000114554	ENSG00000177954
ENSG00000117152	ENSG00000161888	ENSG00000138078	ENSG00000182004	ENSG00000078140	ENSG00000236824
ENSG00000158615	ENSG00000198712	ENSG00000236824	ENSG00000143933	ENSG00000146197	ENSG00000222041
ENSG00000181061	ENSG00000228253	ENSG00000135940	ENSG00000143977	ENSG00000106366	ENSG00000135940
ENSG00000164163	ENSG00000212907	ENSG00000197061	ENSG00000280721	ENSG00000177700	ENSG00000172428
ENSG00000136942	ENSG00000197780	ENSG00000146197	ENSG00000222041	ENSG00000245532	ENSG00000074416
ENSG00000026103	ENSG00000134198	ENSG00000167034	ENSG00000135940	ENSG00000011114	ENSG00000186468
ENSG00000110344	ENSG00000163811	ENSG00000170961	ENSG00000115541	ENSG00000125148	ENSG00000164405
ENSG00000004700	ENSG00000197557	ENSG00000142089	ENSG00000065518	ENSG00000124795	ENSG00000164587
ENSG00000136159	ENSG00000247626	ENSG00000085733	ENSG00000164032	ENSG00000142669	ENSG00000197061
ENSG00000283029	ENSG00000087008	ENSG00000105640	ENSG00000083720	ENSG00000188643	ENSG00000231500
ENSG00000276168	ENSG00000138814	ENSG00000100097	ENSG00000155329	ENSG00000034510	ENSG00000137288
ENSG00000281255	ENSG00000155850	ENSG00000244509	ENSG00000164587	ENSG00000135940	ENSG00000146197
ENSG00000104164	ENSG00000196923	ENSG00000198712	ENSG00000197061	ENSG00000168137	ENSG00000203760
ENSG00000125148	ENSG00000106211	ENSG00000163811	ENSG00000231500	ENSG00000170961	ENSG00000241468
ENSG00000169715	ENSG00000086062	ENSG00000138398	ENSG00000112695	ENSG00000142089	ENSG00000090266
ENSG00000117395	ENSG00000107957	ENSG00000109606	ENSG00000241468	ENSG00000186166	ENSG00000136942
ENSG00000183291	ENSG00000166471	ENSG00000164611	ENSG00000164761	ENSG00000105640	ENSG00000107937
ENSG00000197956	ENSG00000165434	ENSG00000106366	ENSG00000170961	ENSG00000105193	ENSG00000165632
ENSG00000189171	ENSG00000160613	ENSG00000107338	ENSG00000165264	ENSG00000177954	ENSG00000272734
ENSG00000177954	ENSG00000150401	ENSG00000044574	ENSG00000099139	ENSG00000222041	ENSG00000148671
ENSG00000116857	ENSG00000137801	ENSG00000166224	ENSG00000136942	ENSG00000172965	ENSG00000273413
ENSG00000232679	ENSG00000125148	ENSG00000277957	ENSG00000044574	ENSG00000109519	ENSG00000177700
ENSG00000171863	ENSG00000169715	ENSG00000161960	ENSG00000151465	ENSG00000164405	ENSG00000196465
ENSG00000143947	ENSG00000167601	ENSG00000264772	ENSG00000228302	ENSG00000197061	ENSG00000092841
ENSG00000143977	ENSG00000125835	ENSG00000130309	ENSG00000180139	ENSG00000213719	ENSG00000213741
ENSG00000135940	ENSG00000117318	ENSG00000124217	ENSG00000214063	ENSG00000231500	ENSG00000283029
ENSG00000071082	ENSG00000131236	ENSG00000119335	ENSG00000148925	ENSG00000112695	ENSG00000276168
ENSG00000197756	ENSG00000117395	ENSG00000125148	ENSG00000165916	ENSG00000241468	ENSG00000281255
ENSG00000114391	ENSG00000117525	ENSG00000142227	ENSG00000176340	ENSG00000248690	ENSG00000140264
ENSG00000065518	ENSG00000117152	ENSG00000228253	ENSG00000085733	ENSG00000278615	ENSG00000141552
ENSG00000182899	ENSG00000222041	ENSG00000198899	ENSG00000118363	ENSG00000162194	ENSG00000130255

HEK293 freestyle	HEK293T	HUAC	CAP	THP1	Jurkat
ENSG00000113048	ENSG00000075618	ENSG00000189337	ENSG00000155850	ENSG00000146197	ENSG00000168528
ENSG00000144136	ENSG00000116809	ENSG00000172339	ENSG00000002746	ENSG00000104368	ENSG00000183853
ENSG00000125148	ENSG00000187942	ENSG00000121481	ENSG00000132436	ENSG00000074416	ENSG00000137309
ENSG00000198840	ENSG00000169213	ENSG00000182004	ENSG00000148356	ENSG00000006327	ENSG00000104728
ENSG00000135974	ENSG00000229989	ENSG00000152518	ENSG00000125148	ENSG00000125148	ENSG00000213145
ENSG00000104368	ENSG00000143799	ENSG00000236824	ENSG00000198081	ENSG00000138778	ENSG00000257341
ENSG00000136870	ENSG00000169604	ENSG00000177646	ENSG00000180257	ENSG00000154188	ENSG00000125148
ENSG00000165650	ENSG00000178035	ENSG00000114656	ENSG00000117152	ENSG00000173848	ENSG00000167526
ENSG00000169715	ENSG00000170854	ENSG00000187695	ENSG00000163811	ENSG00000140718	ENSG00000125691
ENSG00000261061	ENSG00000109756	ENSG00000122068	ENSG00000173848	ENSG00000154945	ENSG00000198695
ENSG00000154945	ENSG00000111850	ENSG00000170522	ENSG00000148943	ENSG00000248527	ENSG00000177697
ENSG00000101146	ENSG00000169976	ENSG00000153147	ENSG00000125691	ENSG00000070831	ENSG00000185883
ENSG00000177954	ENSG00000070756	ENSG00000151612	ENSG00000259943	ENSG00000126698	ENSG00000128283
ENSG00000181061	ENSG00000136875	ENSG00000134058	ENSG00000084676	ENSG00000131236	ENSG00000121390
ENSG00000235288	ENSG00000139405	ENSG00000224032	ENSG00000170485	ENSG00000169213	ENSG00000279059
ENSG00000163818	ENSG00000136158	ENSG00000197061	ENSG00000115107	ENSG00000122406	ENSG00000104886
ENSG00000174007	ENSG00000140553	ENSG00000146197	ENSG00000197557	ENSG00000117525	ENSG00000197961
ENSG00000113407	ENSG00000267047	ENSG00000048544	ENSG00000178038	ENSG00000197780	ENSG00000161888
ENSG00000113597	ENSG00000126368	ENSG00000277443	ENSG00000164087	ENSG00000160818	ENSG00000142227
ENSG00000122862	ENSG00000006282	ENSG00000106628	ENSG00000163931	ENSG00000143222	ENSG00000210151
ENSG00000283029	ENSG00000183691	ENSG00000013374	ENSG00000170854	ENSG00000117152	ENSG00000144136
ENSG00000276168	ENSG00000176108	ENSG00000235453	ENSG00000243701	ENSG00000143977	ENSG00000134996
ENSG00000281255	ENSG00000169727	ENSG00000203396	ENSG00000114554	ENSG00000115541	ENSG00000260565
ENSG00000280102	ENSG00000079999	ENSG00000169155	ENSG00000113048	ENSG00000270757	ENSG00000068097
ENSG00000274012	ENSG00000099203	ENSG00000207468	ENSG00000111801	ENSG00000144580	ENSG00000173848
ENSG00000257267	ENSG00000198551	ENSG00000175274	ENSG00000112763	ENSG00000181061	ENSG00000157350
	ENSG00000198182	ENSG00000184743	ENSG00000111850	ENSG00000235288	
	ENSG00000142227	ENSG00000185896	ENSG00000104368	ENSG00000144848	
	ENSG00000100029	ENSG00000213928	ENSG00000107957	ENSG00000114023	
	ENSG00000100297	ENSG00000283029	ENSG00000255823	ENSG00000145147	
	ENSG00000189060	ENSG00000276168	ENSG00000160613	ENSG00000078140	
	ENSG00000100226	ENSG00000281255	ENSG00000123374	ENSG00000138814	
	ENSG00000102226	ENSG00000280102	ENSG00000103647	ENSG00000135074	
	ENSG00000157881	ENSG00000274012	ENSG00000259343	ENSG00000164611	
	ENSG00000117318	ENSG00000128944	ENSG00000169715	ENSG00000169045	
	ENSG00000117519	ENSG00000171241	ENSG00000124243	ENSG00000124783	
	ENSG00000265808	ENSG00000261177	ENSG00000154721	ENSG00000112695	
	ENSG00000143401	ENSG00000215067	ENSG00000158164	ENSG00000146263	
	ENSG00000143420	ENSG00000173818	ENSG00000210077	ENSG00000189043	
	ENSG00000173207	ENSG00000206418	ENSG00000116698	ENSG00000256646	
	ENSG00000136628	ENSG00000130816	ENSG00000135940	ENSG00000106144	

Abridged

Table 1. Genes downregulated in fibroblasts by EVs from different cell lines at two different doses.

Cord blood MSC	Bone marrow MSC	Whaton's jelly MSC	BJ-5ta	HUVEC	Panc-1
ENSG00000155363	ENSG00000078808	ENSG00000162408	ENSG00000078808	ENSG00000162576	ENSG00000078808
ENSG00000163155	ENSG00000162576	ENSG00000215695	ENSG00000158109	ENSG00000077549	ENSG00000142961
ENSG00000196754	ENSG00000088280	ENSG00000067704	ENSG00000116273	ENSG00000127483	ENSG00000116704
ENSG00000143341	ENSG00000131238	ENSG00000162909	ENSG00000041988	ENSG00000084636	ENSG00000223745
ENSG00000134318	ENSG00000162407	ENSG00000115295	ENSG00000007923	ENSG00000131238	ENSG00000134250
ENSG00000138073	ENSG00000162434	ENSG00000119820	ENSG00000179051	ENSG00000117385	ENSG00000143179
ENSG00000115207	ENSG00000154027	ENSG00000138061	ENSG00000255275	ENSG00000162407	ENSG00000197965
ENSG00000119820	ENSG00000137962	ENSG00000171150	ENSG00000159423	ENSG00000036549	ENSG00000170385
ENSG00000138061	ENSG00000265972	ENSG00000243244	ENSG00000088280	ENSG00000137962	ENSG00000162909
ENSG00000171150	ENSG00000143398	ENSG00000138069	ENSG00000253368	ENSG00000117620	ENSG00000154429
ENSG00000163719	ENSG00000143543	ENSG00000163235	ENSG00000142733	ENSG00000173218	ENSG00000091483
ENSG00000174738	ENSG00000116604	ENSG00000172071	ENSG00000162419	ENSG00000162745	ENSG00000153187
ENSG00000181555	ENSG00000197965	ENSG00000152256	ENSG00000254553	ENSG00000143162	ENSG00000138080
ENSG00000178467	ENSG00000203705	ENSG00000135919	ENSG00000121900	ENSG00000197965	ENSG00000138002
ENSG00000214706	ENSG00000162909	ENSG00000168958	ENSG00000084070	ENSG00000159388	ENSG00000115295
ENSG00000259976	ENSG00000153187	ENSG00000170275	ENSG00000198815	ENSG00000143479	ENSG00000138061
ENSG00000070476	ENSG00000138073	ENSG00000196345	ENSG00000085998	ENSG00000067704	ENSG00000171150
ENSG00000174928	ENSG00000116016	ENSG00000145022	ENSG00000123472	ENSG00000162909	ENSG00000144043
ENSG00000168924	ENSG00000171150	ENSG00000176095	ENSG00000123473	ENSG00000119280	ENSG00000135632
ENSG00000038219	ENSG00000170340	ENSG00000214706	ENSG00000116212	ENSG00000270106	ENSG00000158158
ENSG00000164402	ENSG00000084090	ENSG00000168297	ENSG00000162402	ENSG00000115884	ENSG00000128641
ENSG00000186314	ENSG00000144021	ENSG00000170871	ENSG00000162601	ENSG00000115295	ENSG00000197121
ENSG00000145861	ENSG00000136715	ENSG00000109743	ENSG00000116641	ENSG00000119820	ENSG00000144476
ENSG00000176783	ENSG00000136536	ENSG00000184178	ENSG00000088035	ENSG00000163171	ENSG00000233608
ENSG00000161011	ENSG00000128641	ENSG00000134851	ENSG00000162434	ENSG00000138061	ENSG00000176095
ENSG00000161010	ENSG00000233608	ENSG00000138758	ENSG00000184588	ENSG00000143889	ENSG00000186792
ENSG00000204227	ENSG00000145022	ENSG00000213949	ENSG00000116704	ENSG00000057935	ENSG00000163320
ENSG00000183826	ENSG00000176095	ENSG00000145730	ENSG00000036549	ENSG00000116016	ENSG00000259976
ENSG00000112146	ENSG00000114480	ENSG00000151304	ENSG00000077254	ENSG00000171150	ENSG00000175166
ENSG00000135317	ENSG00000163320	ENSG00000113083	ENSG00000137942	ENSG00000114978	ENSG00000172061
ENSG00000120910	ENSG00000175166	ENSG00000152377	ENSG00000137962	ENSG00000163162	ENSG00000114473
ENSG00000104517	ENSG00000152492	ENSG00000155508	ENSG00000184371	ENSG00000115641	ENSG00000170871
ENSG00000164961	ENSG00000109171	ENSG00000161011	ENSG00000243960	ENSG00000116044	ENSG00000151552
ENSG00000107186	ENSG00000145216	ENSG00000161010	ENSG00000116455	ENSG00000128641	ENSG00000163697
ENSG00000221829	ENSG00000282278	ENSG00000111799	ENSG00000143079	ENSG00000233608	ENSG00000109171
ENSG00000135049	ENSG00000134851	ENSG00000112249	ENSG00000081026	ENSG00000163719	ENSG00000134851

Abridged

HEK293 freestyle	HEK293T	HUAC	CAP	THP1	Jurkat
ENSG00000162576	ENSG00000078808	ENSG00000107404	ENSG00000162408	ENSG00000142945	ENSG00000143079
ENSG00000157870	ENSG00000160075	ENSG00000189339	ENSG00000179051	ENSG00000184371	ENSG00000143409
ENSG00000142606	ENSG00000116237	ENSG00000162585	ENSG00000077549	ENSG00000143093	ENSG00000143398
ENSG00000116198	ENSG00000090686	ENSG00000171621	ENSG00000127483	ENSG00000197965	ENSG00000143569
ENSG00000157978	ENSG00000117305	ENSG00000054523	ENSG00000158195	ENSG00000143801	ENSG00000143162
ENSG00000142751	ENSG00000117385	ENSG00000264501	ENSG00000123472	ENSG00000182551	ENSG00000197965
ENSG00000084636	ENSG00000162434	ENSG00000251503	ENSG00000185104	ENSG00000144021	ENSG00000203705
ENSG00000164011	ENSG00000137962	ENSG00000175279	ENSG00000137962	ENSG00000065802	ENSG00000091483
ENSG00000126107	ENSG00000184371	ENSG00000198793	ENSG00000117620	ENSG00000228242	ENSG00000035141
ENSG00000258634	ENSG00000134262	ENSG00000120942	ENSG00000116473	ENSG00000145022	ENSG00000163235
ENSG00000143457	ENSG00000117360	ENSG00000116663	ENSG00000163348	ENSG00000010318	ENSG00000172845
ENSG00000143409	ENSG00000143398	ENSG00000116685	ENSG00000143179	ENSG00000144827	ENSG00000135919
ENSG00000143537	ENSG00000116604	ENSG00000142634	ENSG00000162702	ENSG00000065485	ENSG00000237126
ENSG00000162745	ENSG00000152332	ENSG00000215695	ENSG00000159346	ENSG00000163870	ENSG00000163719
ENSG00000159388	ENSG00000143149	ENSG00000255275	ENSG00000159388	ENSG00000175166	ENSG00000157036
ENSG00000133069	ENSG00000143162	ENSG00000159423	ENSG00000133069	ENSG00000168924	ENSG00000145022
ENSG00000143479	ENSG00000197965	ENSG00000090432	ENSG00000143479	ENSG00000134851	ENSG00000010318
ENSG00000203705	ENSG00000151414	ENSG00000088280	ENSG00000143801	ENSG00000163453	ENSG00000168291
ENSG00000143643	ENSG00000176393	ENSG00000011009	ENSG00000180875	ENSG00000037474	ENSG00000081307
ENSG00000170745	ENSG00000117280	ENSG00000204178	ENSG00000143882	ENSG00000184838	ENSG00000069849
ENSG00000138080	ENSG00000203705	ENSG00000130695	ENSG00000138073	ENSG00000113575	ENSG00000175166
ENSG00000176714	ENSG00000162909	ENSG00000090020	ENSG00000119820	ENSG00000273345	ENSG00000170871
ENSG00000013016	ENSG00000115884	ENSG00000142784	ENSG00000143889	ENSG00000161013	ENSG00000134851
ENSG00000115841	ENSG00000138073	ENSG00000142733	ENSG00000171150	ENSG00000137409	ENSG00000163453
ENSG00000143889	ENSG00000115295	ENSG00000117751	ENSG00000116062	ENSG00000198833	ENSG00000138801
ENSG00000171150	ENSG00000119820	ENSG00000130772	ENSG00000035141	ENSG00000172594	ENSG00000145365
ENSG00000170634	ENSG00000171150	ENSG00000197989	ENSG00000158158	ENSG00000078399	ENSG00000189184
ENSG00000239779	ENSG00000035141	ENSG00000060656	ENSG00000144120	ENSG00000122557	ENSG00000037474
ENSG00000115041	ENSG00000115041	ENSG00000121766	ENSG00000172845	ENSG00000155660	ENSG00000153071
ENSG00000082258	ENSG00000144021	ENSG00000254545	ENSG00000123983	ENSG00000105983	ENSG00000113638
ENSG00000153246	ENSG00000163162	ENSG00000134668	ENSG00000135916	ENSG00000164961	ENSG00000213949
ENSG00000155755	ENSG00000115827	ENSG00000160058	ENSG00000131386	ENSG00000122359	ENSG00000164294
ENSG00000135929	ENSG00000116044	ENSG00000254553	ENSG00000182973	ENSG00000107566	ENSG00000086200
ENSG00000224287	ENSG00000128641	ENSG00000162522	ENSG00000178467	ENSG00000108039	ENSG00000013561
ENSG00000279809	ENSG00000178074	ENSG00000160094	ENSG00000016864	ENSG00000151929	ENSG00000113140
ENSG00000182247	ENSG00000233608	ENSG00000134686	ENSG00000168297	ENSG00000150991	ENSG00000112079

Abridged
